# DRACO-STEM: An Automatic Tool to Generate High-Quality 3D Meshes of Shoot Apical Meristem Tissue at Cell Resolution

**DOI:** 10.3389/fpls.2017.00353

**Published:** 2017-03-29

**Authors:** Guillaume Cerutti, Olivier Ali, Christophe Godin

**Affiliations:** ^1^Virtual Plants INRIA Team, UMR AGAP, CIRAD, INRIA, INRAMontpellier, France; ^2^Laboratoire de Reproduction et Développement des Plantes, Université de Lyon, ENS-Lyon, INRA, Centre National de la Recherche ScientifiqueLyon, France

**Keywords:** morphogenesis, shoot apical meristem, triangular mesh, topological optimization, mechanical simulation, python

## Abstract

**Context:** The shoot apical meristem (SAM), origin of all aerial organs of the plant, is a restricted niche of stem cells whose growth is regulated by a complex network of genetic, hormonal and mechanical interactions. Studying the development of this area at cell level using 3D microscopy time-lapse imaging is a newly emerging key to understand the processes controlling plant morphogenesis. Computational models have been proposed to simulate those mechanisms, however their validation on real-life data is an essential step that requires an adequate representation of the growing tissue to be carried out.

**Achievements:** The tool we introduce is a two-stage computational pipeline that generates a complete 3D triangular mesh of the tissue volume based on a segmented tissue image stack. DRACO (Dual Reconstruction by Adjacency Complex Optimization) is designed to retrieve the underlying 3D topological structure of the tissue and compute its dual geometry, while STEM (SAM Tissue Enhanced Mesh) returns a faithful triangular mesh optimized along several quality criteria (intrinsic quality, tissue reconstruction, visual adequacy). Quantitative evaluation tools measuring the performance of the method along those different dimensions are also provided. The resulting meshes can be used as input and validation for biomechanical simulations.

**Availability:** DRACO-STEM is supplied as a package of the open-source multi-platform plant modeling library OpenAlea (http://openalea.github.io/) implemented in Python, and is freely distributed on GitHub (https://github.com/VirtualPlants/draco-stem) along with guidelines for installation and use.

## 1. Motivation

With the ongoing advances in digital microscopy for the monitoring of plant cell development, and the emergence of computational pipelines analyzing such complex 4D data, the understanding of molecular and biophysical processes controlling plant morphogenesis appears closer and closer (Bassel and Smith, 2016). The emerging field of *computational morphodynamics* proposes more and more complex multicellular models simulating growth and shape emergence, with a crucial need for validation (Jönsson et al., 2012).

Image analysis on time-lapse sequences of 3D *z-stacks* (be it confocal laser scanning microscopy or light-sheet microscopy) provides an unprecedented way to access morphometric data of a living tissue at ever-growing spatial and temporal resolutions (Keller, 2013). In plants, such approach generally requires segmenting the cells in membrane-marked images to extract their individual geometry (Fernandez et al., 2010; Federici et al., 2012; Barbier de Reuille et al., 2014; Bassel et al., 2014) and track their shapes in time along with their division events, either automatically (Fernandez et al., 2010) or with the assistance of a human user (Barbier de Reuille et al., 2015). This results in very rich 4D data, and a considerable source of information for validating biological hypotheses transferred into computational models.

However, manipulating voxel-based representations such as 3D images might be inconvenient given the necessary volume of information, and for some applications (visualization, physical simulation) lighter representations are preferred. The geometry of the cells can be represented by their common surfaces, under the form of a (generally triangular) mesh. In the case of biomechanical modeling of the plant tissue, the interactions located at the interfaces between cells are determinant components of the morphogenesis (Hamant et al., 2008), and a representation of the geometry of those interfaces in the most realistic way is essential for the validation of the underlying models (Bassel et al., 2014; Bozorg et al., 2014; Boudon et al., 2015). For approaches based on the classical Finite Element Methods (FEM) the mesh representing the tissue has an additional constraint of containing only regular elements, for a good numerical behavior and valid outputs.

Converting the segmented 3D cell shapes into triangular meshes appears as the best way to obtain those geometries. However, common approaches of isosurface generation such as marching cubes (Lorensen and Cline, 1987) do not create junctions between more than two cells and produce unrealistic, discontinuous, tissue configurations. Non-manifold generalizations have been developed (Hege et al., 1997) but their efficient implementation remains a challenge. Other meshing techniques based for instance on tetrahedral meshes (Shewchuk, 1998) also fail to reconstruct realistic cell shapes and need further processing to be used.

Some other methods have been used to convert tissues into meshes, mostly taking into account the resemblance of plant tissue in the meristematic zone with a Voronoi diagram (Barbier de Reuille et al., 2005) to study the possibility of computing cell geometry as a regular tessellation, which proved to work mostly in 2D (Shapiro et al., 2008). To go to 2.5D (surfacic mesh) (Barbier de Reuille et al., 2015) or to a 3D tessellation (Chakraborty et al., 2013) is possible but results in highly simplified meshes. An optimal conversion that will bridge the gap between experimental acquisitions and computational models is still an open challenge (Bassel and Smith, 2016).

## 2. Algorithms and implementation

**Figure 1:**
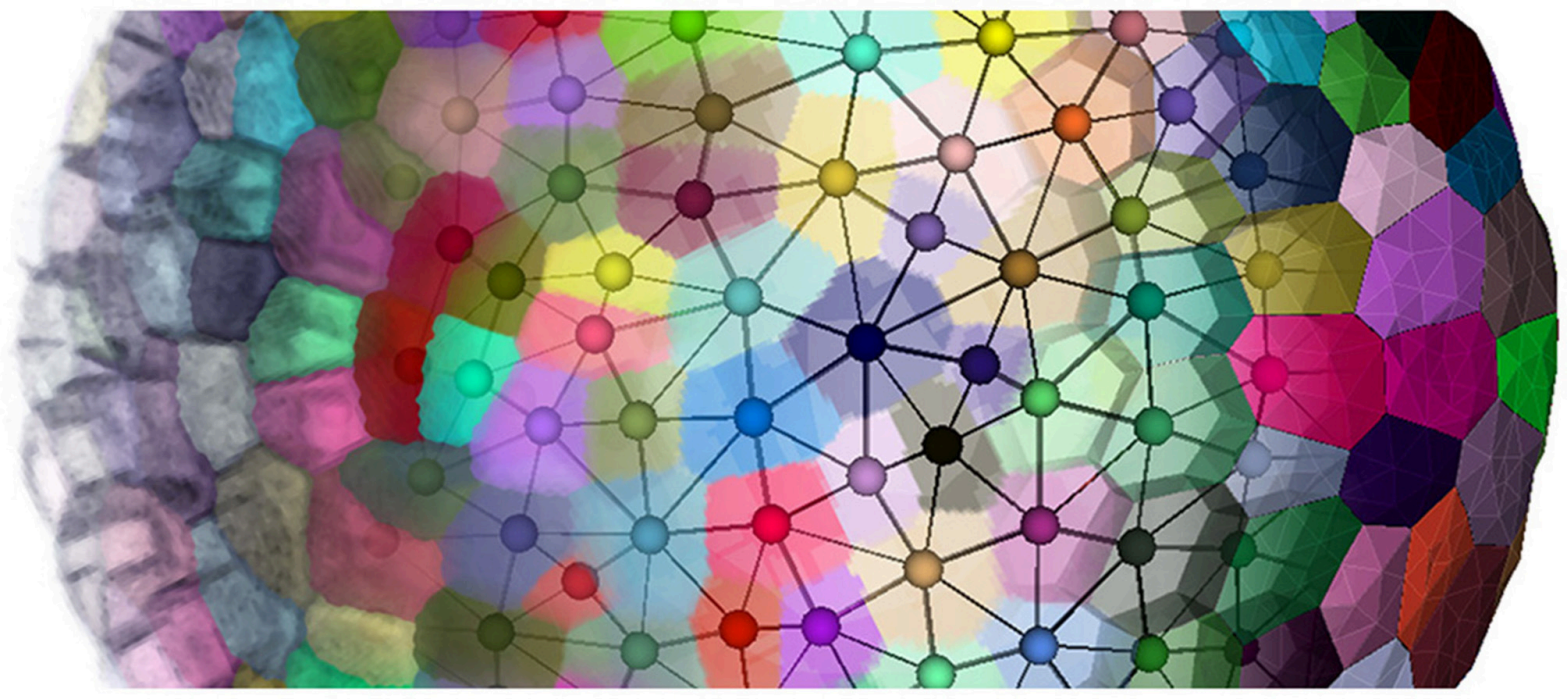
**Going from confocal microscopy image to cell tissue triangular mesh with DRACO-STEM**.

Our objective is to reconstruct 3D, non-manifold, FEM-ready triangular meshes of plant cell tissue from confocal microscopy images, using a dual reconstruction method (as depicted in Figure 1). Our input of the whole pipeline is a segmented shoot apical meristem tissue 3D image stack, obtained using either the MARS-ALT segmentation pipeline (Fernandez et al., 2010), an active region segmentation (Federici et al., 2012) or any 3D watershed (Barbier de Reuille et al., 2015) or convenient 3D segmentation method creating adjacent labeled cell regions.

### 2.1. Definitions and duality

In all the following, we consider that the tissue is a **collection of connected regions** representing the cells. In a first approximation, we assume that these regions form **convex polytopes** (polygons in 2D or polyhedra in 3D) subdividing the space of the tissue. For the sake of simplicity we will first expose our reasoning for the case of a 2-dimensional tissue, where polygonal cells are connected through single edges that represent **cell interfaces**. The vertices where several edges are connected are the **cell corners** where at least three tissue cells meet.

Such a representation falls within the scope of the geometrical notion of **cellular complex** (Agoston, 2005) (Chapter 7: *Algebraic Topology*), naturally used in earlier works to represent cell tissues (Pradal et al., 2009). A cellular complex of dimension *N* is a collection of *n*-dimensional elements (with *n* ≤ *N*) called ***n*-cells**, topologically connected together in such way that:

- Any *n*-cell of the complex has a boundary formed of *k*-cells (*k* < *n*) that are all part of the complex.
- The intersection of two *n*-cells is either empty or *k*-cells (*k* < *n*) belonging to both their boundaries.

**Figure 2:**
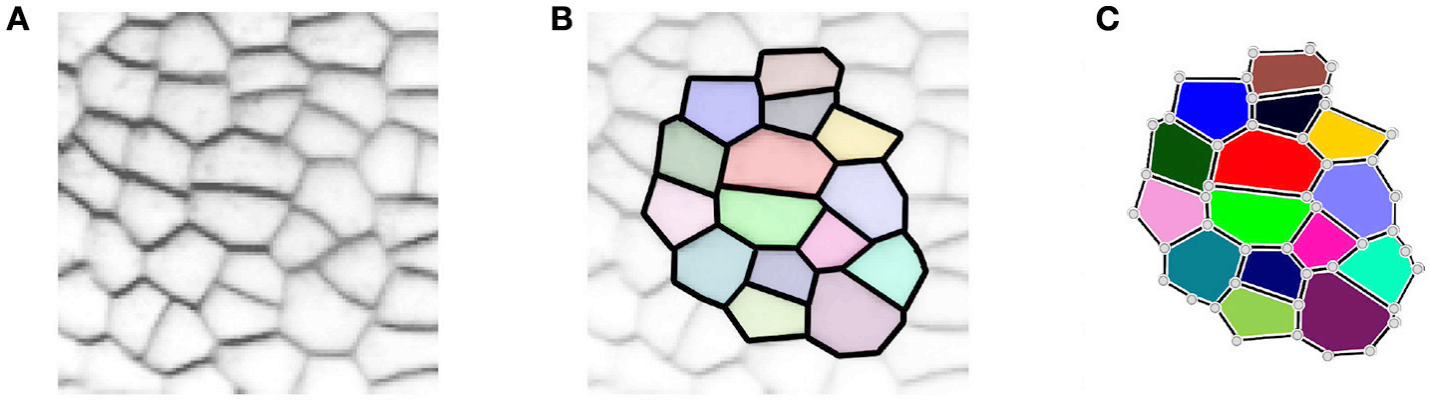
**A two-dimensional cell tissue (A)** where cells are seen as a collection of connected polygons **(B)** that can be represented as a cellular complex **(C)**.

In 2D, the tissue is then represented by a set of vertices, edges and convex polygons (respectively 0, 1 and 2-cells) where polygons intersect only at their boundary edges, and edges at their boundary vertices. A simple vision of how meristematic plant tissue can be represented as a cellular complex is given in Figure 2.

We add an even more restrictive hypothesis for cellular complexes representing plant tissue geometry, stating that only **exactly 3** polygonal 2-cells (representing the cells of the tissue) can meet at vertices of the complex. From a biological point of view, this could be interpreted as the assumption that a plant cell always shares a wall (at least a tiny portion) with all of its neighbors, so that the interface between two cells could not be reduced to a single vertex. In the tissues, ambiguous junctions implying 4 or more cells can occur, but they are not preserved in time and the evolution of adjacency provides information on which cells were neighbors.

Through the notions of shared boundaries and interfaces, such cellular complex representation also provides information on the **adjacency** between tissue cells. An interface between two 2-cells defines an adjacency link between the tissue cells they represent, and the vertices where three 2-cells meet can be seen as **triangles of adjacency** linking the three concerned tissue cells. Considering the adjacency between cells is another way of looking at the tissue structure and the **adjacency object** formed by adjacency links and triangles constitutes a complementary view to the **geometry object**.

**Figure 3:**
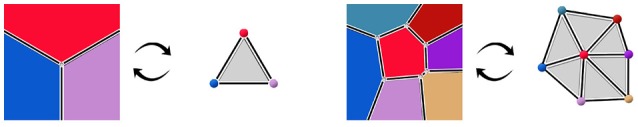
**Dualization of cellular complexes representing cells the geometry of tissue cells into simplicial complexes representing their adjacency**.

More precisely, we consider that those two objects are the **dual** of each other. The notion of duality refers to the idea that lowest dimension elements of a topological object correspond the highest dimension elements in its dual, and *vice versa*. For instance, 2-cells, the highest dimension elements in the geometry complex, representing tissue cells, are converted into points in the dual. Interfaces between tissue cells (edges, or 1-cells in the geometry) are converted into adjacency edges (dimension 1), and cell corners (vertices, or 0-cells in the geometry) correspond to triangles linking tissue cells in the adjacency (dimension 2). The vertices representing tissue cells in the adjacency object can for instance be placed at the center of the region of their corresponding cell. If we consider adjacency edges connected to only one adjacency triangle to be dual to infinite cell interfaces, this produces a reversible mapping between the two objects, illustrated in Figure 3. Consequently, we consider that the adjacency complex contains the same topological information as the geometry complex, and it might be a more suitable representation of the tissue depending on the application.

The vertices where cells meet in the geometry complex are mapped in the dual adjacency object to triangles which are **2-simplices**. A *n*-simplex is a *n*-dimensional element that is the convex hull of its *n*+1 vertices, generalizing the notion of triangle to any dimension. The consequence is that the dual of the cellular complex of geometry constitutes a **simplicial complex** of adjacency, a special case of cellular complex where all *n*-cells are *n*-simplices. In 2D, simplicial complexes are triangulations, and the **Delaunay triangulation** of the cell center points constitutes an example of adjacency simplicial complex (De Berg et al., 2008) (Chapter 9: *Delaunay Triangulations*). It is a complex of triangles built on a set of vertices such that no vertex lies inside the circumscribed center of any triangle; its dual geometry is the **Voronoi diagram** of the center points, where cells are the volume of space closer to the point they represent than to any other.

In this setting, our problem consists then of reconstructing the cellular complex representing the geometry of the tissue given a **segmented image** as input. We consider segmented images as connected labeled regions, each label representing one tissue cell. Such an image encompasses all the information on the geometry of the tissue under the form of a connected grid of pixels, but the abstraction which is necessary to extract the cellular complex is not obvious to perform. An image directly defines a cellular complex in which 2-cells are pixels, and converting it into a complex with tissue cells as highest dimension elements requires a merging process in which it is difficult to ensure that topological properties will be preserved.

However, it is much easier to perform an abstraction of the adjacency relationships from the segmented image. A lot of useful information can be extracted from this pixel-based representation: cell center points can be computed as the center of mass of the regions, neighbor pixels of different labels can create adjacency links between the corresponding cells, and pixel squares containing at least three different labels can be extracted to define adjacency triangles.

**Figure 4:**
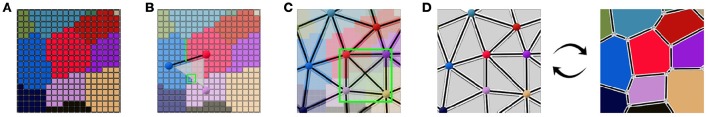
**Abstracting an adjacency simplicial complex from a 2D segmented image: cells as connected regions of labeled pixels (A)**, extraction of adjacency simplices from pixel neighborhoods **(B)**, the set of adjacency simplices not necessarily forming a simplicial complex **(C)**, and the reconstructed valid simplicial complex converting directly into the geometry cellular complex through dualization **(D)**.

This collection of simplices does not necessarily form a simplicial complex, as shown in Figure 4C where 4 triangles are overlapping in the ambiguous area, thus not partitioning the space. But if we use them to reconstruct a simplicial complex of adjacency (therefore forcing a choice in the ambiguous junctions), computing its dual is a straightforward way to obtain an approximated geometry of the tissue as a cellular complex. This is the key idea that drives our reconstruction method. The Figure 4 illustrates this process of extracting adjacency simplices from pixel data to reconstruct a simplicial complex for which the dualization is direct.

**Figure 5:**
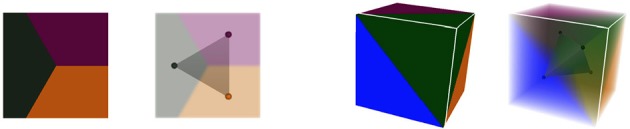
**Duality in different dimensions: in 2D, three regions of adjacent geometry create a triangular simplex of adjacency in the dual domain, as in 3D the dual of 4 adjacent regions forms a tetrahedral simplex**.

These notions of cellular complexes of geometry and their dual simplicial complexes of adjacency extend very well to 3D. Tissue cells are then represented as polyhedra sharing polygonal interfaces, bounded by edges, and cell corners where **exactly 4 cells** meet. Consequently, those points convert to **adjacency tetrahedra** in the dual domain, 3-simplices, that define a simplicial complex. Cell interfaces still correspond to adjacency links, and interface edges to adjacency triangles, ensuring that cells share a surface of wall with their neighbors. Here as well, the **Delaunay tetrahedrization** is the dual of the 3D Voronoi diagram. The Figure 5 shows this extension of 2D duality to 3D.

3D segmented image stacks are defined over a grid of voxels that makes neighborhood between labeled regions more complex. But all adjacency simplices can still be extracted considering neighborhood relationships between voxels of different labels, up to the adjacency tetrahedra detected in voxel cubes where at least 4 different labels meet. Constructing a simplicial complex of such tetrahedra and computing its dual produces a 3D geometry simplicial complex. Then, the only step left is to triangulate the faces of such structure to obtain a **3D triangular mesh** of the tissue, the very object we aim to produce.

### 2.2. Data structure

As we just saw, the 3D triangular meshes and polyhedral complexes representing the tissue geometry, as well as the adjacency simplicial complexes, may all be represented by the topological structure of cellular complex. In the case of tissue meshes, the 3-cells (elements of dimension 3) of this complex are the actual cells of the tissue, whereas in adjacency complexes, they correspond to tetrahedra linking 4 tissue cells adjacent to each other in the tetrahedrization.

**Figure 6:**
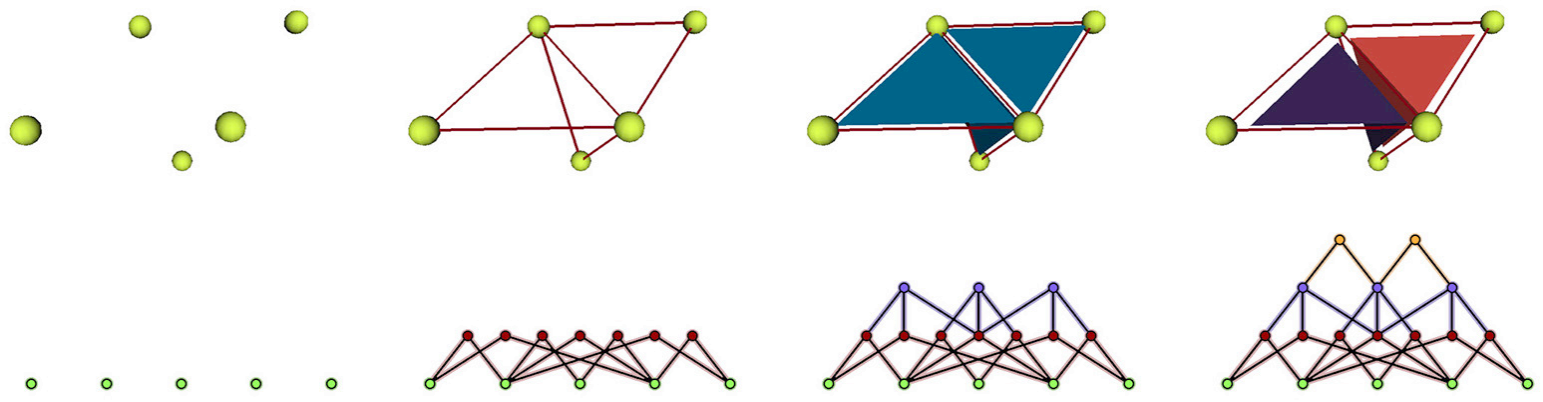
**Representation of a mesh containing 2 cells defined by 3 triangular faces as an incidence graph: 3D mesh on the first line, incidence graph representation on the second; the edges defining the triangles are linked to their two extremities, the triangles to their respective edges, and the cells to their boundary triangles**.

We implement cellular complexes as incidence graphs, a graph-like boundary representation in which the nodes are the 0, 1, 2, and 3-cells of the complex (respectively vertices, edges, faces, and polyhedra) and the links define the boundary relationship between elements of consecutive dimensions. For instance an element of dimension 1 will generally be linked to two elements of dimension 0, the two vertices that define it as an edge. The nodes of the graph can bear additional properties to make the data structure a rich representation of annotated cell complexes. An example of such a representation used in a triangular mesh is given in Figure 6.

All the connectivity information between the elements is included in the graph structure, a great advantage to perform topological operations. Another advantage is that generally, the dualization operation applied on an incidence graph simply consists in flipping over the graph: all the elements are preserved, as well as their topological relationships, but their dimensions are swapped (elements of dimension 3 converting into elements of dimension 0 in the dual graph, dimension 2 into dimension 1, and so on).

In such topological representations, the geometry of the structure relies on the spatial positions affected to the vertices (elements of dimension 0) that will define the shapes of all higher dimension elements. The topological relationships constraint the overall shape of the object, but in the end the local appearance of its components depends entirely on the geometry. In particular, to obtain triangular meshes usable for FEM applications, it is required to adjust the positions of the vertices with a high concern for regularity of the triangles.

### 2.3. Methods

The DRACO-STEM algorithm aims at computing a 3D triangular mesh of the tissue by the dualization of a simplicial complex of adjacency, and the triangulation and optimization of the resulting polyhedral geometry. As stated earlier, adjacency simplices can be extracted from the segmented image. However, due to segmentation errors, local noise, or converging regions, they do not necessary form a simplicial complex (see **Figure 4C**). Some might intersect, or even be included in another, or holes may exist inside the tissue. To perform the dual reconstruction, it is then necessary to build a valid simplicial complex that includes as many image-extracted simplices as possible, which is the idea behind our method.

**Figure 7:**
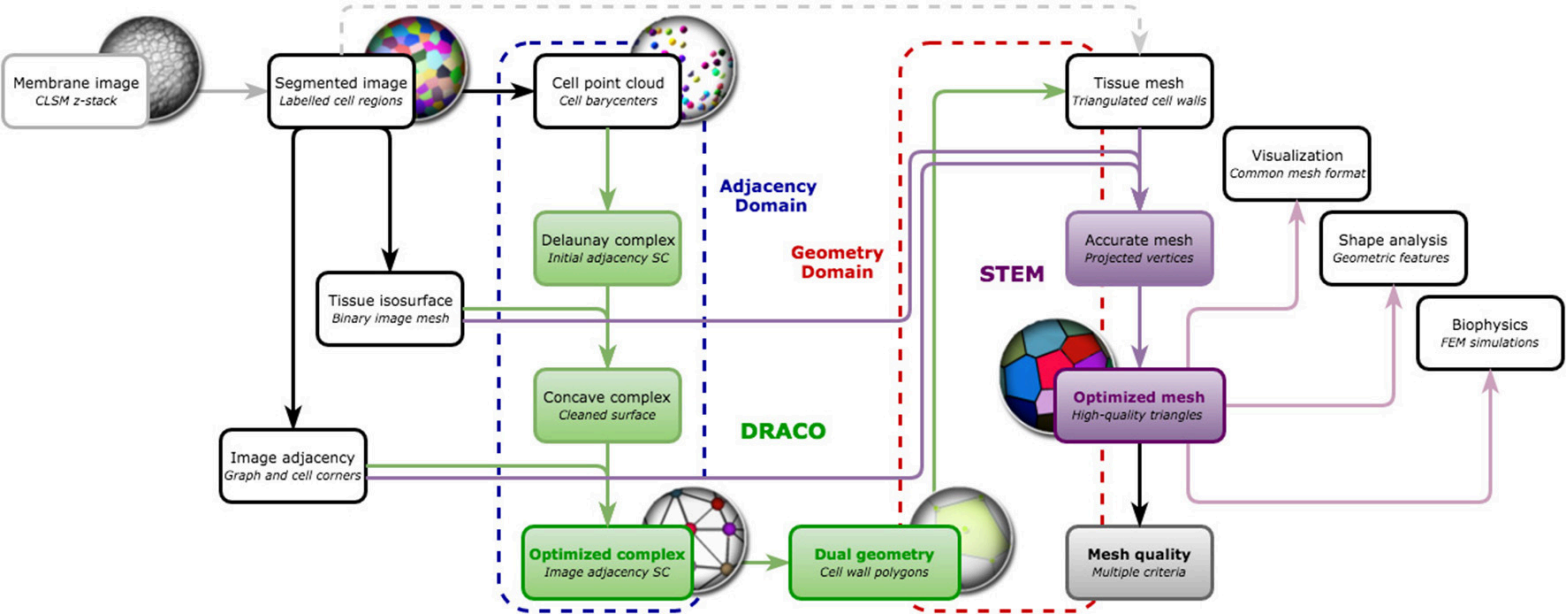
**Overview of the DRACO-STEM components: DRACO (→) creates an adjacency complex by the image-based optimization of the Delaunay complex of cell barycenters and generates the dual geometry complex, and STEM (→) optimizes a triangular tissue mesh, coming from DRACO or not, along several meristem tissue specific criteria and estimates its quality**. The algorithmic components operate either on adjacency complexes (- - - ) in the case of DRACO or geometry complexes (- - - ).

The mesh construction algorithm can then be split into two independent steps illustrated in Figure 7:

- The first one (DRACO) starts by extracting cell neighborhood relationships from the segmented image to reconstruct a simplicial complex of cell adjacencies that optimally matches them. This adjacency complex is either optimized from a valid initial guess for the whole tissue, or, if limited to the outermost cell layers, constructed by aggregation of simplices extracted from the image. The resulting complex can directly be dualized into a valid topological representation of the tissue. Its geometry is defined by setting cell corners to their position in the image, and possibly triangulating the cell interfaces.
- The second step (STEM) starts from such a 3D triangular mesh of the tissue, obtained from the DRACO algorithm, or from another topologically accurate meshing method [interfaces of tetrahedral image mesh (Shewchuk, 1998) or decimated multi-label marching cubes mesh (Hege et al., 1997)]. The algorithm performs a specific optimization of the geometry of the mesh to improve simultaneously the regularity of the triangles and the shape of the cells while keeping the topological and geometrical consistency with the segmented image in the best compromise possible.

The proposed mesh generation pipeline relies on a set of original mesh processing algorithms operating on cellular complexes and trying to optimize both their topology and their geometry along several criteria, through energy minimization. Such complex optimization processes are necessary to obtain a faithful mesh of the original tissue, with convex polygonal cell interfaces and triangles regular enough to be used as an input to simulation methods.

#### 2.3.1. Dual reconstruction by adjacency complex optimization (DRACO)

The first component (DRACO) works on the dual adjacency object to later reconstruct a geometry, using the method detailed in Cerutti et al. (2015). This idea initially comes from the fact that the geometry of the meristematic tissue forms a regular tessellation (Shapiro et al., 2008) that strongly evokes a Voronoi diagram of the cell centers. However, the tissue presents strong local anisotropy in the shapes of its cells that can not be well approximated by a Voronoi diagram. This anisotropy can be explained by the local mechanical constraints on the cells and by the directed growth and division processes that create highly variable shapes. The very isotropic Voronoi diagram would end up creating walls between cells that are not actual neighbors.

Since this tessellation is the dual of a simplical complex (the Delaunay tetrahedrization) in which tetrahedral units define adjacencies between cells by their edges, the fact that the Voronoi diagram creates wrong walls can be related to the fact that some adjacency edges are wrong in the Delaunay complex. The Delaunay criterion creates edges between non-adjacent cells of the tissue. In consequence, if we correct this adjacency simplicial complex so that it fits the actual adjacencies observed in the tissue, the resulting dual geometry will be a much better approximation of the cell walls.

**Figure 8:**
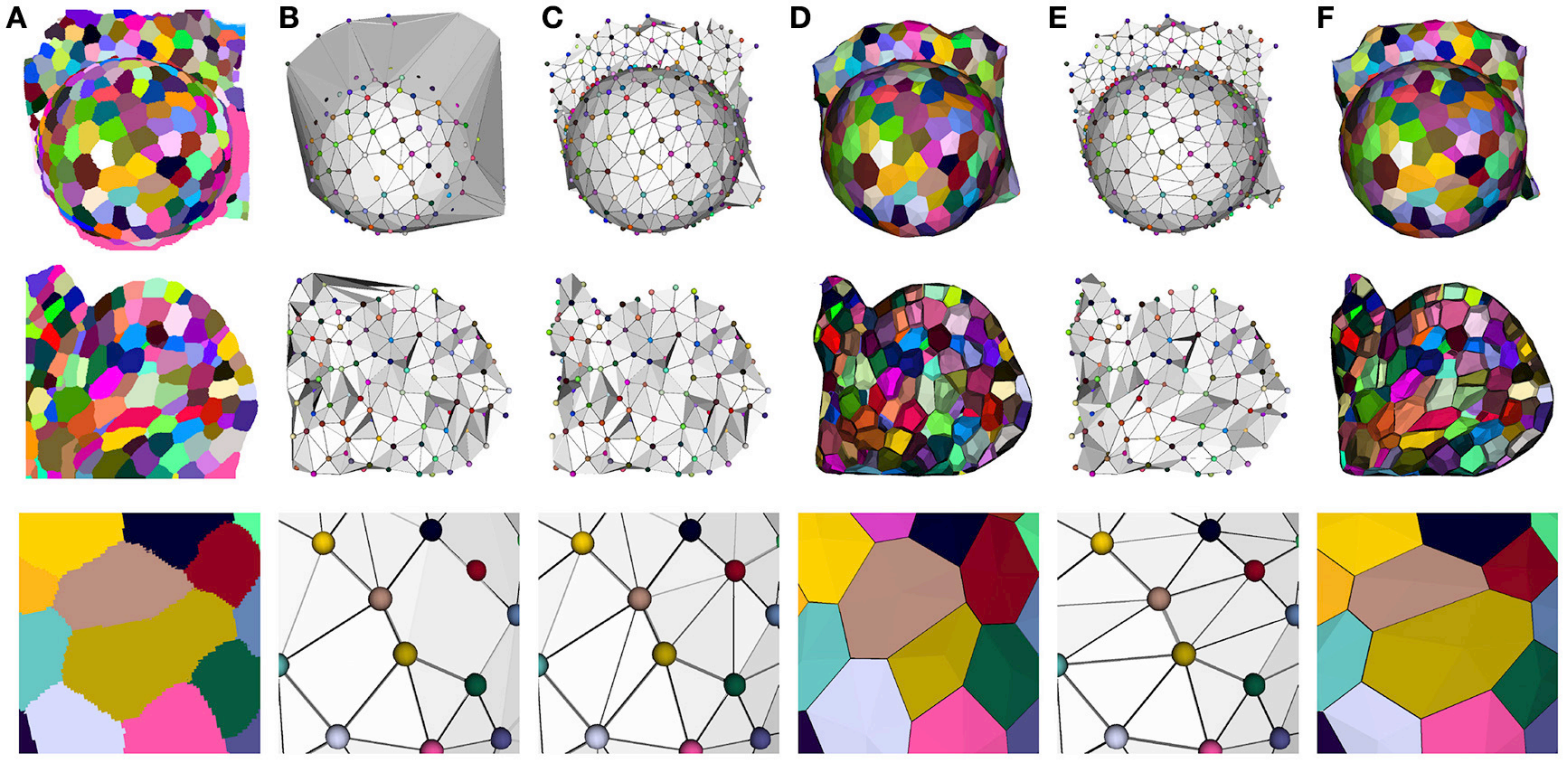
**Constructing a dual geometry by the optimization of the cell adjacency simplicial complex: initial segmented image (A)**, Delaunay tetrahedrization **(B)** and cleaned adjacency complex **(C)**, Voronoi diagram obtained by the dualization of the cleaned Delaunay complex **(D)**, optimized adjacency complex **(E)** and dual tissue reconstruction **(F)**. The cell complexes in **(D,F)** are triangulated for visualization.

Our optimization method reflects this idea, and takes the Delaunay tetrahedrization of the cell centers as a valid starting point. This complex is then optimized by a two step process to fit the adjacencies extracted from the image. The first one consists in getting rid of the excessive triangles due to the constraint of convexity of the Delaunay complex (see Figure 8B). This is done by successively removing exterior triangles that either cross the surface of the tissue, present too long edges or form flat tetrahedra (or slivers).

Then the actual tissue adjacencies are optimized following an iterative process performing local topological operations (triangle swaps and edge removals, Shewchuk, 2002) in order to minimize an energy functional. This energy defined on the adjacency simplicial complex T is composed of several terms to take into account the adjacencies extracted from the segmented image S, the prior knowledge about the number of possible neighbors for a cell, and the regularity constraints preventing elongated tetrahedra so that distant cells do not end up as neighbors. Each term is weighted by a coefficient so that its relative influence can be fine-tuned:

(1) $$\begin{array}{l} E(T,S)=\omega_{image}\;E_{image}(T,S)\;+\;\omega_{prior}\;E_{prior}(T)\\ \;+\;\omega_{regularity}\;E_{regularity}(T) \end{array}$$

The content and formulation of those three energy components can be found in Section 1.2 of Supplementary Material. The energy minimization takes the form of an iterative simulated annealing process, with successive temperature cycles, high temperatures allowing non-optimal transformations, in order to reach non-trivial optimal configurations when the temperature lowers. This method shows a great improvement in the adjacency estimation, recovering nearly 90% correct adjacency links when the Delaunay complex only reached 76% (Cerutti et al., 2015).

**Figure 9:**
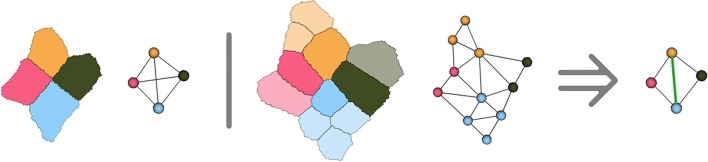
**Illustration in two dimensions of the ambiguous junction problem : when more than 4 cells meet, the simplicial complex hypothesis forces to make a choice concerning cell adjacency, that might only be verified by looking at the further evolution of the ambiguous junction**.

However, due to the hypotheses made on cell adjacency detailed in Section 2.1, the method might create incorrect adjacencies in the ambiguous cell junctions where 5 cells or more come close together. Considering that the adjacency complex forms a tetrahedralization of the cell centers structurally forces the method to get rid of the ambiguity by making a decision on which 4 cells are actually going to be adjacent in the tissue development. Any choice would be correct based on the image, since in these cases all the cells will have voxels in contact, and the decision will then be mostly made based on geometry and number of neighbors. As shown in Figure 9, the only way to ensure that the decision made at this point is right would be to compute cell lineages between consecutive time points to know which groups of daughter cells will be adjacent.

The dualization of the resulting adjacency complex provides a simple polyhedral representation of the cells. An important point for the accuracy of this polygonal mesh is to determine the position of its vertices, and we chose, as in a Voronoi diagram, to place them at the center of the circumscribed sphere of their dual adjacency tetrahedron. However, since the Delaunay constraint no longer holds, we had to force them to remain inside their tetrahedra by projection on the nearest face, to avoid geometrical artifacts (face intersection notably), using a process detailed in Section 1.3 of Supplementary Material. The result of this step is a very simple tissue mesh composed of polyhedral cells but reflecting faithfully the topological structure of the tissue with accurate cell walls, as shown in Figure 8.

#### 2.3.2. Layer dual reconstruction

In the general case, the adjacency object formed using the adjacency simplices extracted from the image does not constitute a valid simplicial complex. But it is possible to take advantage of the specific layered structure of the meristem tissue to reconstruct only the two outermost layers of cells. The epidermal layer L1 and the one right underneath L2 are very well separated and constitute independent sets of cells. This means that if we consider the simplicial complex of adjacency of L1 and L2 cells, the set of triangles linking only L1 cells forms a simplicial complex, as well as set of triangles linking only L2 cells. Consequently, the adjacency complex between L1 and L2 cells will only contain tetrahedra either composed of a triangle of L1 cells and one L2 cell, a triangle of L2 cells and one L1 cell, or two L1 cells and two L2 cells.

This is a very strong prior information, not valid further down in the tissue, that can be used to create a valid simplicial complex by aggregation of tetrahedra that correspond to the structure of these tissue layers. To do so, we added a way of iteratively constructing the complex from a set of candidate tetrahedra extracted from the segmented image. Each tetrahedron is assigned a weight taking into account the distances between the cells it links and the area of the wall they share. The process starts from the tetrahedron achieving the best weight and adds to the complex its neighbors that do not create intersections, selecting the highest weights and unambiguous configurations first. The added tetrahedra are placed into a queue to continue the exploration, until no new candidate can be added.

**Figure 10:**
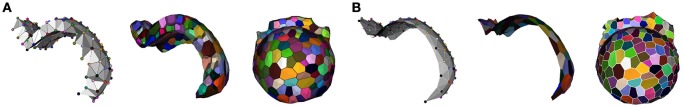
**Layer reconstruction using adjacency complexes: L1-L2 tetrahedrization and its dual 2-layer 3D geometry (A)** and L1 triangulation and its dual 2.5D geometry **(B)**.

The resulting complex is a single layer of tetrahedra that makes it possible to reconstruct accurately the L1 and L2 cells by dualization. The same aggregation process can also be used to reconstruct a single layer of cells by building a simplicial complex made of triangles representing the cell adjacencies inside the L1 or L2 layer. This allows us to reconstruct a surfacic 2.5D polygonal mesh of the cell layer. Figure 10 gives an illustration of these two alternative methods.

#### 2.3.3. SAM tissue enhanced mesh (STEM)

A triangular mesh obtained out of the DRACO pipeline may be too coarse and present too many irregular triangles for a use in FEM-based simulations. It is therefore required to provide a way of enhancing such structures. The second stage of our mesh generation pipeline consists then of a multi-objective optimization algorithm for non-manifold triangular meshes, based on the methodology described in Cerutti and Godin (2015). It takes as input a triangular mesh such as the one obtained by triangulating the DRACO geometry (either by Delaunay, or more simply placing an additional vertex at the center of each cell interface, a process referred as “Star” triangulation from now on) or by another coarse, topologically accurate meshing method.

The mesh is first passed through optional pre-processing steps to refine it (and make it more precise), enhance its topological properties and its local accuracy:

- Simple triangle split refinement (creating 4 identical triangular faces for each input face).
- Isotropic remeshing with target edge length for global refinement (Botsch and Kobbelt, 2004).
- Surface vertex projection onto the surface of the meshed object (extracted as a binary isosurface).
- Cell corner identification and positioning based on the positions extracted from the segmented image.

**Figure 11:**
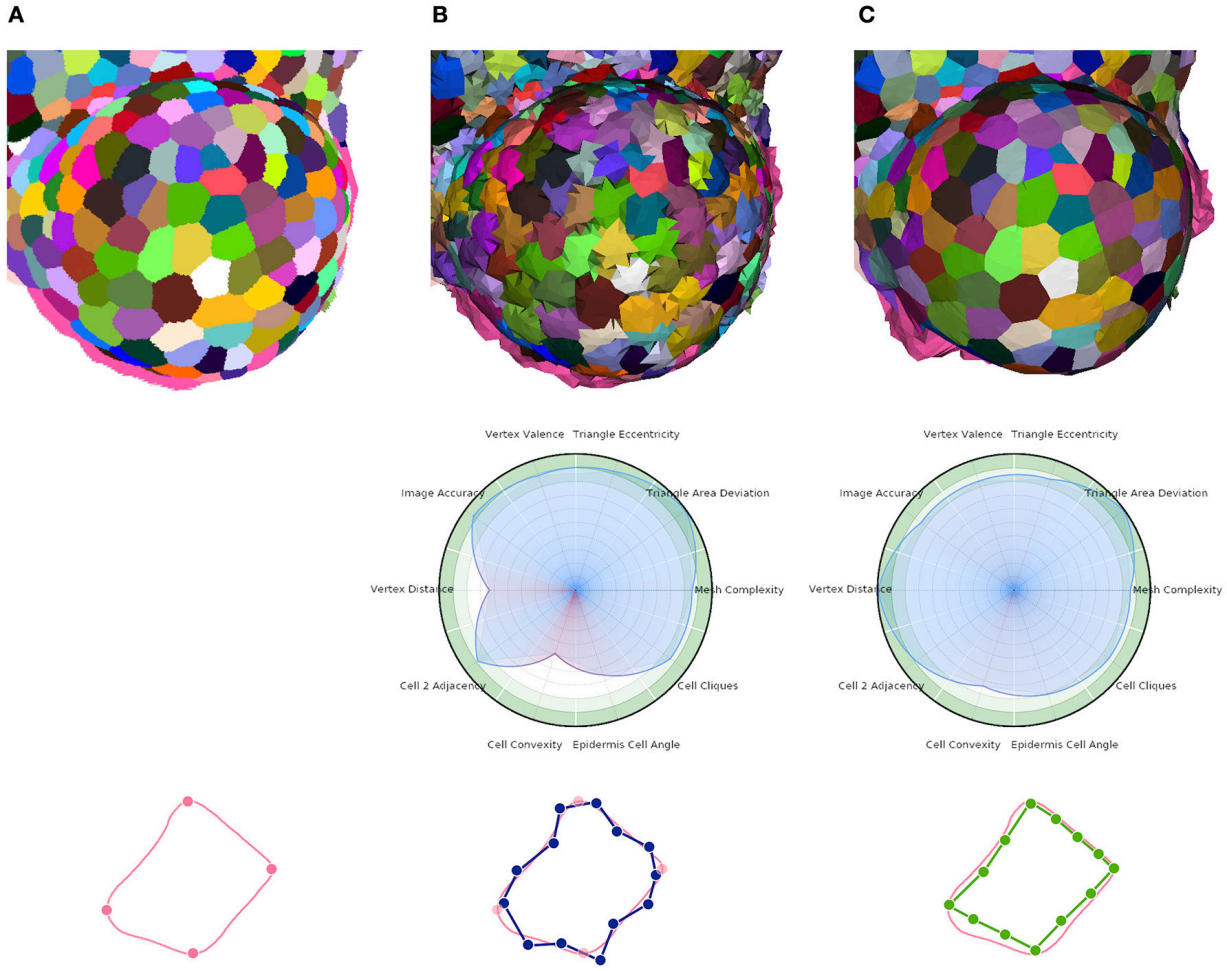
**Multi-criteria quality estimation applied on two tissue meshes**. Original segmented tissue image **(A)**, coarse 3D triangular mesh obtained from interfaces of Delaunay refinement tetrahedra (IDRA) **(B)** and enhanced tissue triangular mesh optimized using the STEM algorithm (IDRA-STEM) **(C)**, and a schematic representation of application of STEM on a cell interface contour.

Then, the core optimization process can be applied. Here again, it is implemented as an iterative energy minimization process performing local operations to improve several criteria simultaneously. This time, the local operations are vertex shifting (relocation of the vertex points in a small sphere around their current position) and interface triangle edge flips. The energy minimized by the process has the same form as Equation (1), with an image attachment term tying vertices to actual cell interfaces, a shape prior term producing polygonal flat cell walls, and a regularization term ensuring regularly shaped triangles and isotropic vertex neighborhoods (more details in Section 1.5 of Supplementary Material). The Figure 11 gives an example of the application of this tissue specific mesh enhancement algorithm.

#### 2.3.4. Quantitative quality estimation

The quality of a mesh is not an unambiguously defined concept, and depends a lot on the application intended for the considered object. No unique, objective quality measure exists, it has to be defined to meet the expectations of the end-user. In our context of reconstruction of a biological entity from an input image with the perspective of usability in FEM simulations, the notion of quality spreads over several aspects:

- **Prior consistency**: the resulting mesh should be consistent with what we expect from the tissue structure we are reconstructing, based on the hypotheses we made on tissue structure. On the geometry side, we expect to have convex cells with convex polygonal facets, and on the topology side, we assume that the mesh should be a cellular complex dualizable into a simplicial complex.
- **Image consistency**: since the mesh is based on a real acquisition, it should also correspond as well as possible to the segmented image used as input (which we consider a valid representation of the tissue, as far as possible) both geometrically (the shapes and contours of the cells) and topologically (adjacency relationships between cells).
- **Intrinsic regularity**: finally, considering the goal of using the mesh for FEM suimulations, the regularity of the triangular elements of the mesh should be as high as possible for sake of numerical robustness, both geometrically (regularity and homogeneity of the triangles) and topologically (valence of the vertices).
- **Complexity**: in addition, to ensure a fast visualization and a reasonable computation time when running simulations, this multi-faceted quality should be achieved using as few triangles as possible, and we consider therefore the complexity of the mesh (in terms of number of elements) as a component of its global quality.

To obtain objective measures defining the quality of the 3D triangular meshes resulting from the DRACO-STEM pipeline, we defined a set of normalized estimators covering all these different aspects. Many of these measures have their values comprised between 0 and 1, but some do not, and we chose to normalize them by a handset “optimal” value to make a simultaneous visualization and visual comparison easier. We end up with 10 quality estimators defined as following:

- **Cell convexity** (prior global geometry): estimated for each cell in the mesh as the ratio between its volume and the volume of its convex hull, and averaged across the mesh. *(no normalization)*.
- **Epidermis cell angle** (prior local geometry): computed at each surface cell junction as the absolute difference of the angles made by cells at the junction with the theoretical value of 120°, and averaged across the mesh. *(normalized by an angular deviation of 30 degrees, measured negatively from 1)*.
- **Cell cliques** (prior topology): percentage of the cell junctions (mesh vertices neighboring at least 4 cells, or 3 on the surface) where strictly more than 4 (respectively 3) cells meet. *(no normalization, measured negatively from 1)*.
- **Image accuracy** (image global geometry): estimated for each cell in the mesh as the Jaccard index (overlap measure) of the voxels of corresponding regions in the segmented image and in the voxelized mesh image, and averaged across the mesh. *(no normalization)*.
- **Vertex distance** (image local geometry): computed for each cell junction vertex in the mesh as the distance to its corresponding point in the image, averaged across the mesh. *(normalized by* $\frac{\sqrt{3}}{4}$*, length of a voxel diagonal in an image of typical 0.25*μ*m resolution, measured as a reciprocal)*.
- **Cell 2-adjacency** (image topology): computed as the Jaccard index (overlap measure) of the cell adjacency edges extracted from the image and those in the dual adjacency mesh. *(no normalization)*.
- **Triangle area deviation** (intrinsic global geometry): estimated as the standard deviation of the mesh triangles. *(normalized by* $\sqrt{2}$ *times the average area of the triangles, measured negatively from 1)*.
- **Triangle eccentricity** (intrinsic local geometry): measured using the average eccentricity of the mesh triangles, computed using the sum of the sinuses of the triangle. *(normalized by* 0.5*, measured negatively from 1)*.
- **Vertex valence** (intrinsic topology): the average absolute difference between the number of neighbors of mesh vertices and their optimal value (6 inside interfaces). *(normalized by 6, measured negatively from 1)*.
- **Mesh complexity** (global complexity): estimated using the average number of triangles necessary to represent one cell. *(normalized by a reasonable number of 152, leading to a suitable total number of faces for finite element models of a 1,000-cell tissue, and corresponding to a good triangulation of the space-filling truncated octahedron, measured as a reciprocal)*.

Defined this way, the quality of a mesh can conveniently be visualized on a circular plot (or spider-web) as the one shown in Figure 11 and provide an immediate visual comparison of the pros and cons of a meshed tissue. In this example, the first mesh has strong defects on the local cell geometry (Vertex distance) as well as on the consistency of cell geometries with biological prior (Cell convexity and Epidermis cell angle). The second mesh largely corrects this defects but actually does so by sacrificing a bit of regularity (Triangle eccentricity) and faithfulness to the image (Image accuracy).

Such a quantitative quality analysis is a precious tool to ensure the structures that are produced by the method will meet the requirements of the application it is intended for. Providing specific quality estimators along with the mesh generation algorithms is a way to guarantee the reproducibility of the presented results.

### 2.4. Implementation and visualization

The DRACO-STEM algorithms, as well as the data structure used to represent cellular complexes are supplied as packages of the open-source plant modeling library OpenAlea (Pradal et al., 2008, 2009). This library is designed as a middleware providing tools and components to build dynamic systems implementing plant models. It is developed in Python language and is available on the GitHub software sharing platform.

**Figure 12:**
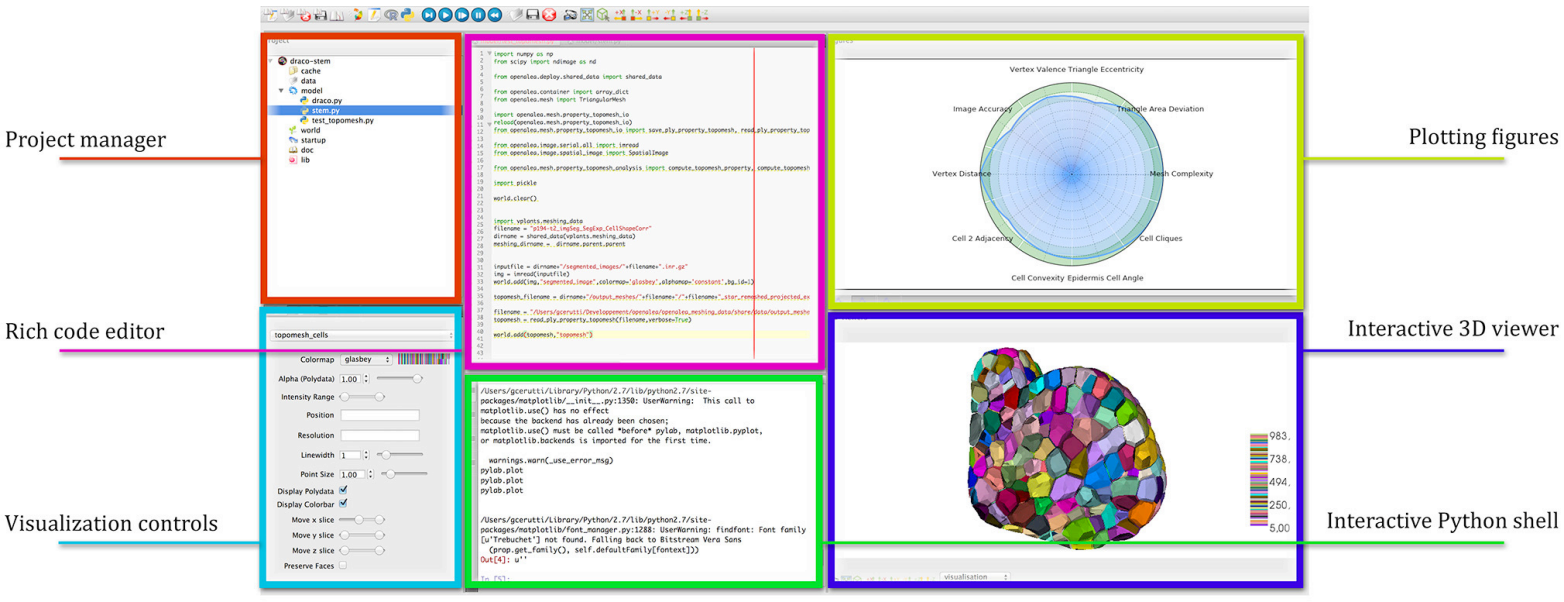
**Interaction with a cellular complex mesh in the TissueLab model development platform**.

OpenAlea comes with a multi-paradigm development platform called OpenAleaLab which allows modelers to integrate different sources into a unique customizable environment (Coste et al., 2014). In particular, specific component related to cell tissue modeling have been developed into a particular instance of the development environment (TissueLab) to provide easy visualization and manipulation of cell tissue structures. This way, cellular complexes representing the tissue can be visualized in interaction with tissue images, along with other useful visual components (plots, interactive console, code editor). Figure 12 shows an example of the integration in the OpenAleaLab platform.

Using the DRACO-STEM algorithms within the OpenAlea framework is rather simple as it is integrated to work on the same data structures and with the same philosophy as the rest of the library. A specific class named DracoMesh is used to perform the optimization and dualization and is instantiated using an image as argument. Then three main functions applied to this object reflect the different steps of the process (Delaunay complex creation, adjacency complex optimization and dual reconstruction) and allow to manipulate parameter values. An example of a program generating a 3D tissue mesh using DRACO-STEM is given in Table 1 of Supplementary Material.

## 3. Results and discussion

### 3.1. Mesh generation results

We applied our evaluation method to triangular tissue meshes generated from a dataset of segmented tissue images. This dataset was composed of two time-series of a floral meristem at early developmental stages, containing respectively 3 and 10 time points. Each image contains in average around 1,000 cells, and offers a good diversity of surface curvature, and quite variable cell shapes. Using different meshing techniques and evaluating the resulting structures using the same quantitative criteria provided us with extensive measures of the pros and cons of each method, and is the best way to assess which constitutes the best compromise. The methods we evaluated correspond to those implemented in the library we propose, and all constitute ways to reconstruct a topologically accurate tissue reconstruction:

- **Interfaces of Delaunay refinement tetrahedra** (IDRA): mesh obtained by converting the image into a coarse tetrahedral mesh using Delaunay refinement (Shewchuk, 1998), and keeping only the triangles belonging to tetrahedra of different labels.
- **Interface enhanced tissue mesh** (IDRA-STEM): same method as the previous one, except that the mesh is optimized using the STEM algorithm.
- **Voronoi Diagram of the cell centers** (Voronoi (Star)): obtained by dualization of the Delaunay tetrahedrization of the cell center points, in which the interfaces are triangulated simply by placing a vertex at the interface center and linking all interface edges to it (star triangulation).
- **Dual reconstruction using the optimized cell complex** (DRACO (Star)): obtained by dualization of the simplicial complex of adjacency optimized from the Delaunay one using the DRACO algorithm; interfaces triangulated using the star triangulation.
- **Dual reconstruction enhanced tissue mesh** (DRACO-STEM): same method as the previous one, except that the star interface mesh is remeshed locally, projected on the tissue surface and optimized using the STEM algorithm.

Concerning the other methods that could have been included, we chose to discard the standard 3D Marching Cubes (Lorensen and Cline, 1987) as well as its implementation in MorphoGraphX (Barbier de Reuille et al., 2015) because the produced meshes do not contain adjacency connections between more that two cells, and fail at representing accurately the topology of cell junctions. The Generalized Marching Cubes (Hege et al., 1997) would correct this specific problem, and so could probably the adaptive tessellation method (Chakraborty et al., 2013), but they are not available to our knowledge in any freely available package, and could therefore not be tested.

The results of the evaluation of the retained methods are presented in Table 1 summing up the average value and standard deviation obtained for all the different quality estimators on the tested dataset.

**Table 1:**
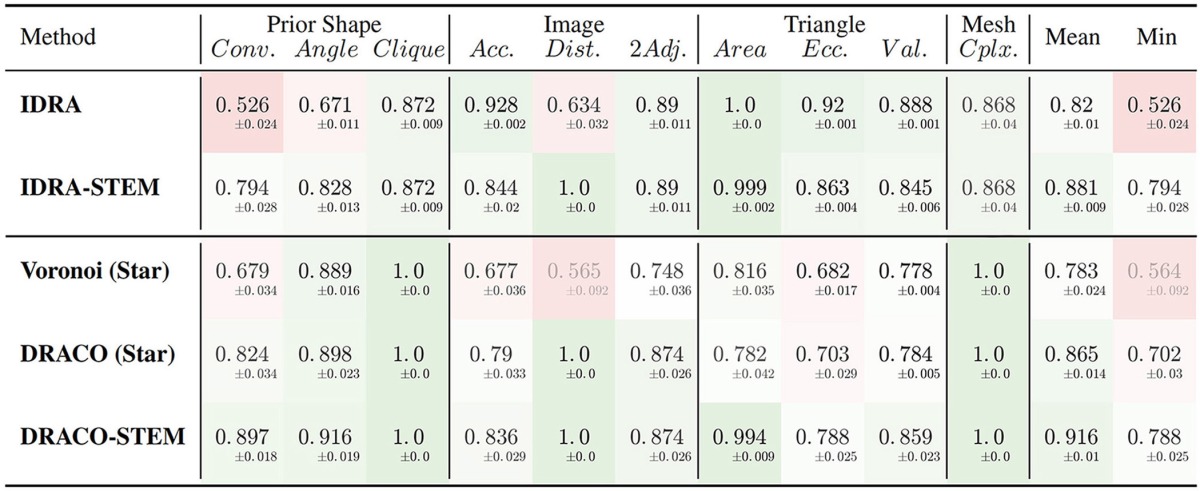
**Average quality measures on meshes obtained with different generation methods: dual reconstructions from Delaunay or optimized adjacency complexes, interfaces of Delaunay refinement tetrahedra and their STEM enhanced versions**.

The first conclusion to draw from these results is the clear improvement provided by the adjacency complex optimization method we introduced. When comparing the results of the meshes obtained from Delaunay (Voronoi (Star)) and the ones from the optimized complex (DRACO (Star)), triangulated using the same method, there is a clear improvement in the image consistency criteria (image accuracy: 0.677 → **0.790** [ANOVA, *p* < 0.001] and cell adjacency: 0.748 → **0.874**, [*p* < 10^−7^]). The cell shapes are also more realistic, with a better convexity (0.679 → **0.824** [*p* < 10^−7^]). The application of DRACO therefore results in a striking improvement of the overall mesh quality (0.783 → **0.865** on average [*p* < 10^−7^]).

The other notable information is the benefit provided by our specific mesh optimization procedure STEM. Applied either on the IDRA or the DRACO meshes, this optimization leads to a significant increase in the average quality (0.82 → **0.881** and 0.865 → **0.916** respectively [*p* < 10^−7^]) and maybe more importantly in the minimal value of the estimators, meaning that no aspect of the quality is left apart. This increase concerns first the shapes of the cells (notably cell convexity and cell angle estimators) but globally preserves the other values in the same time. The only exception is the decrease of image accuracy (0.928 → **0.844** [*p* < 10^−7^]) and triangle regularity (0.92 → **0.863** [*p* < 10^−7^]) in the case of IDRA meshes, which comes from the fact that the initial meshes have very regular triangles and are globally consistent with the image, but with very noisy cell boundaries as visible in Figure 11B, and on the vertex distance estimator. The application of STEM corrects the latter at the expense of decreasing the regularity of the mesh elements and the volumetric consistency with the image as in Figure 11C.

Concerning the comparison between IDRA and DRACO meshing methods, their differences before any optimization appear clearly in our quality estimators. IDRA provides meshes with a very high regularity that globally fit very well the cell regions in the image, but with oscillating to spiky boundaries that make the unusable as such. DRACO meshes do not show the same regularity, in particular with the star triangulation and no remeshing (triangle eccentricity: **0.920** | 0.703, image accuracy: **0.928** | 0.790 [*p* < 10^−7^]). However in terms of the local shape of the cell interfaces and precision at the level of the cell junctions, they are clearly superior (cell convexity: 0.526 | **0.824**, vertex distance: 0.634 | **1.0** [*p* < 10^−7^]).

The necessary application of STEM, which was designed to draw meshes toward a good compromise, tends to even things up on several aspects. IDRA-STEM and DRACO-STEM meshes have close values of image consistency indicators (image accuracy: **0.844** | 0.836 [*p* > 0.1], cell adjacency: **0.890** | 0.874 [*p*≃0.05]) even if the regularity of the mesh elements remains clearly lower (triangle eccentricity: **0.863** | 0.788 [*p* < 10^−7^]). But the difference on the cell shape estimators (cell convexity: 0.794 | **0.897** [*p* < 10^−7^]) and the resulting average quality (0.881 | **0.916** [*p* < 10^−7^]) demonstrates that the DRACO-STEM algorithm provides an excellent compromise between all the desirable properties of a 3D tissue mesh.

**Figure 13:**
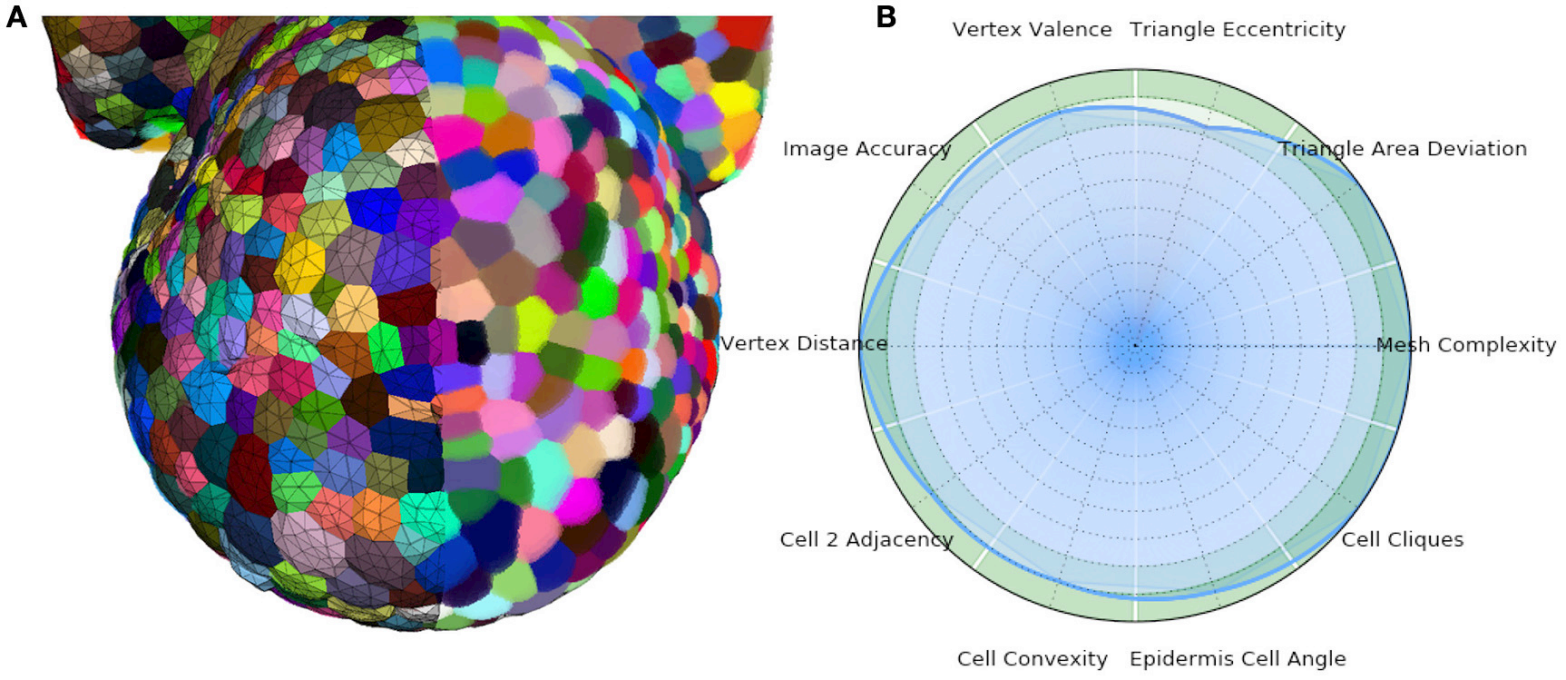
**3D triangular mesh generated using the DRACO-STEM pipeline on a segmented flower meristem image (A)** and its multi-criteria quality evaluation **(B)**.

In the end, the result of this pipeline is a mesh that corresponds as well to the image as its optimized tetrahedral mesh conversion, but with much better properties regarding the shapes and arrangement of its cells, and a regularity and size that makes it adequate for a use in biophysical simulations. The Figure 13 shows an example of such a mesh structure along with its quality evaluation.

### 3.2. Geometrical and biological information projection

The mesh generation algorithms come with a complementary mesh processing library that allows in particular to compute geometrical properties on a tissue mesh. The cellular complex structure is defined in such way that several properties can be assigned to its elements of different dimensions. For instance, one can compute the volumes of the cells and the area of the interfaces, and both will be stored simultaneously in the structure along with other properties.

In particular it is possible to estimate curvature information (mean curvature, Gaussian curvature, principal directions, curvature tensor) on the surface of the mesh, measures that may be difficult to obtain using another tissue representation, but can be easily estimated on a surfacic triangular mesh (Theisel et al., 2004). Such intrinsic geometric property of the tissue may be of great interest put in perspective with other biological measures obtained at the surface of the tissue.

**Figure 14:**
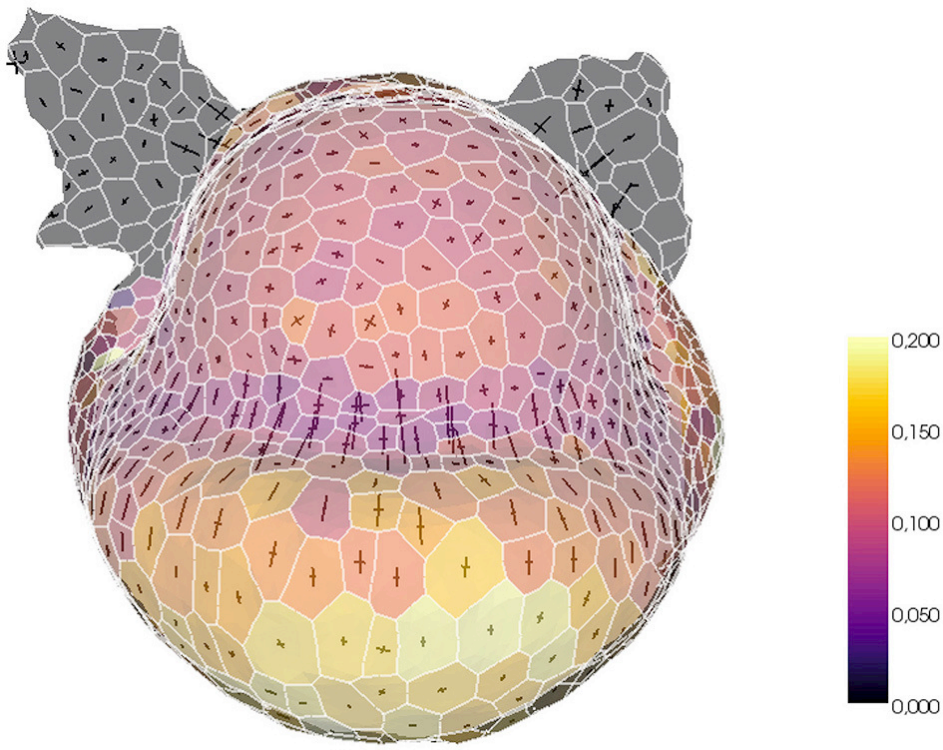
**Example of biological data projection: visualizing volumetric cell growth rates (colorscale) and principal curvature tensors on a mesh of L1 cells of a flower meristem**.

The tissue structure can also be used to project external information onto a convenient spatialized visualization. For example, a fluorescence-based biological signal (hormone concentration or genetic expression) quantified from a nuclei image can be turned into a property of the cells of the tissue and visualized with the same tools. Or, given a sequence of images of the same organ with the cells tracked in time, the mesh can be used as a support for the visualization of growth rates and directions. The tissue structure constitutes then a very useful tool for the visualization and exploration of morphogenesis processes, as illustrated in Figure 14.

### 3.3. Biomechanical simulations

Triangular tissue meshes produced using the DRACO-STEM pipeline have been used in the context of biomechanical simulation of cellular growth. In plants, growth can be assimilated to the yielding of cell walls under Turgor-induced stresses. It can be formalized through differential equations accounting for this yielding behavior and the mechanical equilibrium of the whole structure. Numerically, these equations can be simulated using the Finite Element Method (FEM). This approach suffers from two major bottlenecks: first, the subtle and complex shapes of organic tissues require a high quality meshing pipeline in order to generate biologically relevant structures. Second, stability of the numerical integration schemes depends heavily on the regularity in shape and size of the meshing triangles. In this perspective, tools such as the STEM optimization where the intensity of regularization can be fine tuned can really help to achieve stable and accurate simulations.

**Figure 15:**
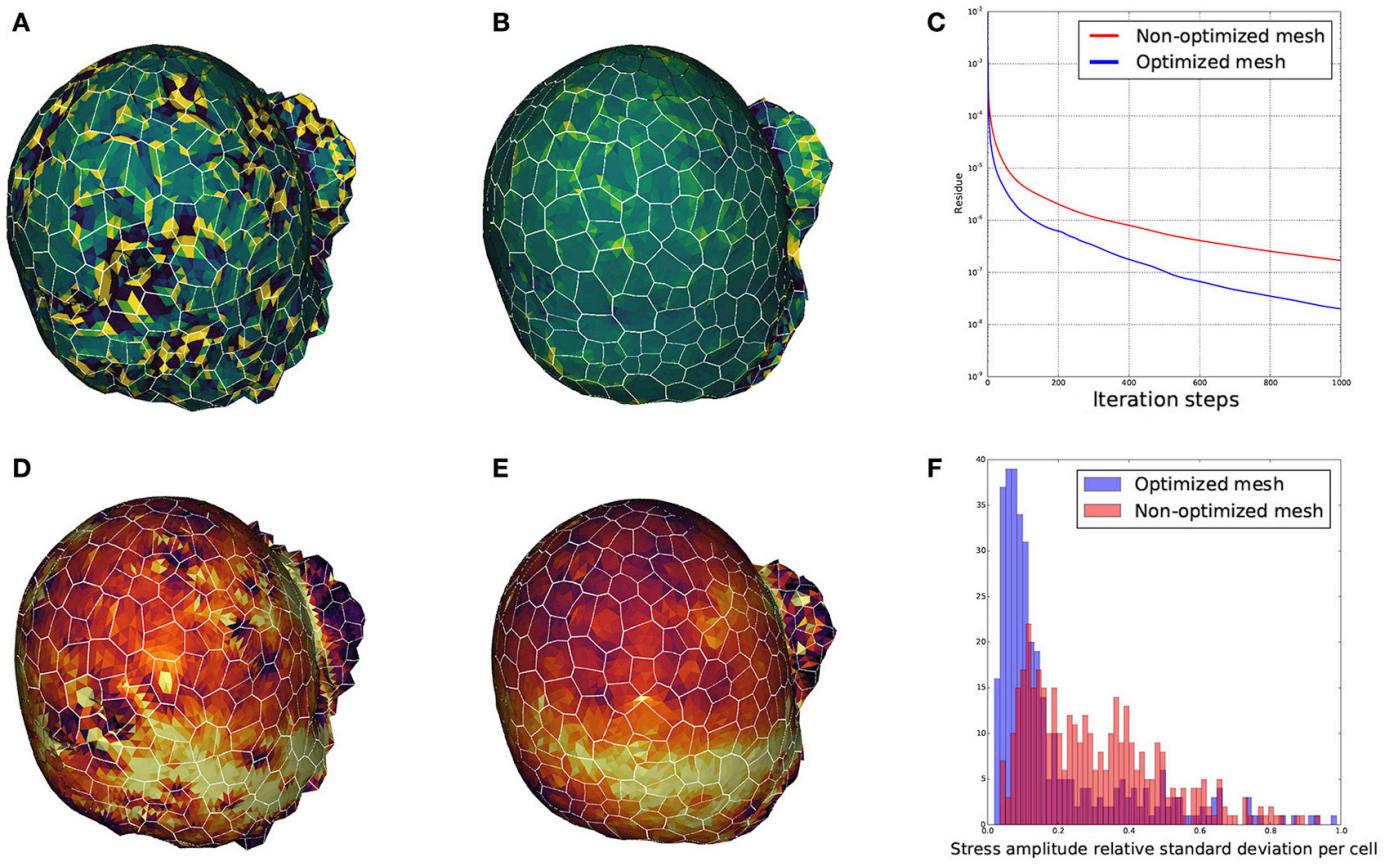
**Influence of mesh optimization on biomechanical simulations: comparisons between the local gaussian curvature on a non optimized DRACO mesh (A)** and an optimized DRACO-STEM mesh **(B)**, the convergence speed (error as a function of iteration steps) in an implicit Euler integration scheme in both cases **(C)**, and estimation mechanical stress amplitudes respectively **(D,E)**, and comparison of their cellwise standard deviations **(F)**.

We wanted to ensure that the triangular meshes produced by DRACO were suitable for such applications and that the STEM component was pertinent in this context. Therefore, to estimate the influence of regularization on mechanical simulations, we meshed the same segmented tissue using DRACO with and without STEM optimization to compare the behavior of the numerical solver (Allard et al., 2007) and the outputs of the mechanical simulations in each case. A quick look at the smoothness of each of the meshed structures reveals much bigger local variations of the Gaussian curvature in the non-optimized structure (Figure 15A) than in the optimized one (Figure 15B). During the simulation, the local quality of the mesh is expected to affect greatly the numerical convergence of the system toward a mechanical equilibrium. We then compared the precision of estimation of the mechanical equilibrium by monitoring the residue of the Euler implicit integration scheme. Figure 15C shows that the remaining residue is almost ten times smaller with the optimized structure compared to the more irregular one (after 1,000 iterations steps only), meaning that the optimization of the mesh makes the simulation converge much faster.

Finally, we looked at the mechanical stress amplitude distribution throughout the structure. Figures 15D,E show that, though the average stress amplitude is roughly the same for both structures, bigger variations between neighboring finite elements appears on the non-optimized structure. This is also exposed on Figure 15F where the distribution of standard deviations for the stress amplitudes within cells are displayed. While the histogram issued from the optimized structure is rather pinched a little bit below 10%, the one from the non-optimized structure is more widespread, reinforcing the idea that non-optimized structures generate highly fluctuating and less accurate stress fields.

These results validate if necessary the fact that the 3D triangular meshes constructed using our pipeline can be used directly for advanced biomechanical simulations. They constitute robust geometries on which finite element based methods can be applied safely. The study also underlines the major importance of the second geometry optimization step in order to get a better performance of the numerical solver and obtain more precise and trustworthy results out of physical simulations.

### 3.4. Conclusions

This computational tool provides meshing methods needed for biophysical simulations of growing SAM tissue on real-life data. The DRACO-STEM algorithm, together with 3D image processing methods provides an integrated pipeline to convert a living tissue acquired in fluorescent microscopy into a single ready-to-use data structure, combining both geometry and topology of a multi-layered tissue. Through the application of commonly defined file standards (Krupinski et al., 2015) already included in the proposed library, the produced meshes may be used in any applicative context of morphodynamic modeling.

The perspectives opened for biomechanical simulations of organ development in plants in particular are extremely promising and the first tests performed on shoot apical meristems show the pertinence of the proposed approach. The extension to other plant tissues presenting similar characteristics (approximately convex cells with flat interfaces), e.g., young leaves and root apical meristems, should in principle be possible, as these are the main assumptions made in our method. The proposed pipeline proves to supply modelers with accurate tissue geometries robust enough for numerical solvers. The availability of such a generic tool represents a key step in the development of accurate and compelling computational biophysical models of plant morphogenesis.

## Author contributions

GC and CG conceived and designed the computational framework. GC developed and implemented the mesh generation algorithms, and performed tests on segmented images. GC documented and released the code on GitHub. OA developed the mechanical framework and performed tests on the produced meshes. GC, OA, and CG wrote the manuscript.

## Funding

This work was supported by the Human Frontier Science Program (HFSP) research grant RGP0054-2013, by the Computational Biology Intitute (IBC) and by the IPL Morphogenetics.

### Conflict of interest statement

The authors declare that the research was conducted in the absence of any commercial or financial relationships that could be construed as a potential conflict of interest.
