# Supplementary material for: DRACO-STEM: An Automatic Tool to Generate High-Quality 3D Meshes of Shoot Apical Meristem Tissue at Cell Resolution
Source: Front Plant Sci. 2017 Mar 29;8:353. doi: 10.3389/fpls.2017.00353 (PMC5372818; doi:10.3389/fpls.2017.00353)
Supplement: Supplementary file 1 [file Presentation1.pdf]

DRACO-STEM : an automatic tool to generate high-quality 3D meshes of meristem tissue at cell resolution

Guillaume Cerutti [1,*], Olivier Ali [1,2], Christophe Godin [1]
*Correspondence:
Guillaume Cerutti
guillaume.cerutti@inria.fr

1 TECHNICAL DETAILS

In this section, we explain more thoroughly some of the mathematical and algorithmic tools used in the DRACO-STEM pipeline. Most of them concern the optimization of a cellular complex, either for the representation of the adjacency (simplicial complex) or for the representation of the tissue geometry (triangular mesh). Consequently we will first introduce notations describing such objects, and in a second time detail the specific methods used in the algorithm. More complete details can be found in previous publications (Cerutti and Godin, 2015; Cerutti et al., 2015)

1.1 Notations

As explained in Section 2.2, a cellular complex is represented in our framework by the data structure of **incidence graph** where the n-cells of the complex are nodes of the graph, and the edges correspond to a boundary relationship between n-cells and $n-1$-cells. For a cellular complex of dimension 3, we will consider four distinct **sets of elements**:

- $\mathcal{C}_0$ corresponding to the vertices or 0-cells of the complex
- $\mathcal{C}_1$ corresponding to the edges or 1-cells of the complex
- $\mathcal{C}_2$ corresponding to the faces or 2-cells of the complex
- $\mathcal{C}_3$ corresponding to the polyhedral cells or 3-cells of the complex

The edges of the graph define relationships that associate an element of dimension n with its boundaries of dimension $n-1$. We define explicitly this **boundary relationship** by a function that defines the boundary of an element of $\mathcal{C}_n$ as a set of elements of $\mathcal{C}_{n-1}$, which can also be split into 3 sets:

- $\mathcal{B}_1$ assigning a set of vertices of $\mathcal{C}_0$ that form the boundary of an edge of $\mathcal{C}_1$
- $\mathcal{B}_2$ assigning a set of edges of $\mathcal{C}_1$ that form the boundary of a face of $\mathcal{C}_2$
- $\mathcal{B}_3$ assigning a set of faces of $\mathcal{C}_2$ that form the boundary of a cell of $\mathcal{C}_3$

For instance, any triangular face $t \in \mathcal{C}_2$ will have its boundary defined as $\mathcal{B}_2(t) = \{e_1, e_2, e_3\}$ with $e_1, e_2, e_3 \in \mathcal{C}_1$. This boundary relationship $\mathcal{B}_n$ defines also a converse **region relationship** $\mathcal{R}_{n-1}$ listing, for an element of dimension $n-1$, all the elements of dimension n for which it constitutes a boundary:

$$\forall c \in \mathcal{C}_n,\ \mathcal{R}_n(c) = \{c' \in \mathcal{C}_{n+1} \mid c \in \mathcal{B}_{n+1}(c')\}$$

The notion of boundaries (and regions) can be extended to more than one dimension by mere transitivity, and noted $\mathcal{B}_n^k$ with $\mathcal{B}_n^1 = \mathcal{B}_n$:

$$\forall c \in \mathcal{C}_n, \mathcal{B}_n^k(c) = \bigcup_{b \in \mathcal{B}_n(c)} \mathcal{B}_{n-1}^{k-1}(b)$$

This also defines two different **neighborhood relationships** between elements of the same dimension n. Either we consider the elements of dimension n that share a same boundary of dimension $n-1$ or the elements of dimension n that share a same region of dimension $n+1$. Those two relationships are respectively written as $\mathcal{N}_n^-$ and $\mathcal{N}_n^+$.

$$\mathcal{N}_n^-(c) = \{c' \in \mathcal{C}_n \mid \mathcal{B}_n(c) \cap \mathcal{B}_n(c') \neq \emptyset\} \qquad \mathcal{N}_n^+(c) = \{c' \in \mathcal{C}_n \mid \mathcal{R}_n(c) \cap \mathcal{R}_n(c') \neq \emptyset\}$$

All these relationships, that allow to navigate through the structure and define local measures, are illustrated in Figure 1.

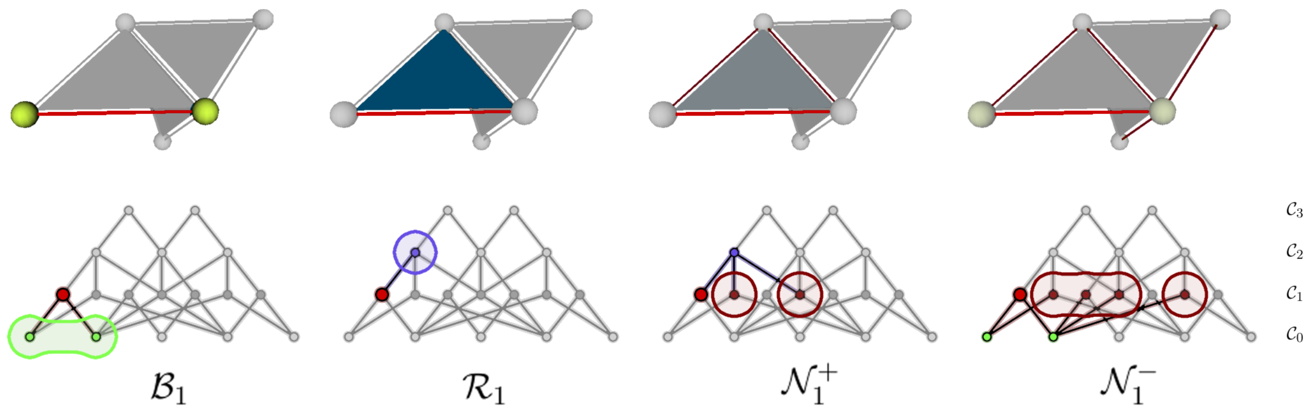

Figure 1. Representation of the topological relationships between elements of a cellular complex implemented as an incidence graph: boundary relationship, region relationship, ascending and descending neighborhood relationships.

Taken alone, the elements of the incidence graph and the boundary relationships only define the topological organization of the structure. The geometrical information that completes its definition is specified by a 3D spatial position assigned to each vertex of $\mathcal{C}_0$. The geometry of all higher dimension elements is then determined by the positions of the underlying vertices:

$$\mathcal{P} = \{\mathcal{P}(v), v \in \mathcal{C}_0\}$$

1.2 Energy definition for adjacency complex optimization

In the DRACO process, the essential step consists in optimizing a simplicial complex of cell adjacency by an iterative process of energy minimization. The energy that is minimized by the algorithm is defined over a simplicial complex $\mathcal{T}$ with respect to a segmented image $\mathcal{S}$ that the complex aims to represent. The formula of the energy given in Section 2.3.1 but recalled here for convenience:

$$E(\mathcal{T}, \mathcal{S}) = \omega_{image} E_{image}(\mathcal{T}, \mathcal{S}) + \omega_{prior} E_{prior}(\mathcal{T}) + \omega_{regularity} E_{regularity}(\mathcal{T}) \tag{1}$$

As evoked in Section 2.1, a set of adjacency simplices can be extracted from the segmented image $\mathcal{S}$. For instance tetrahedra of adjacency can be detected by looking for cubes $\cube$ formed by 8 voxels that contain at least 4 different labels. Considering all these possible cubes, we obtain a set of adjacency simplices written:

$$T_{\mathcal{S}} = \left\{\{c_1, c_2, c_3, c_4\} \mid \exists \cube \in \mathcal{S}, c_1, c_2, c_3, c_4 \in \cube\right\}$$

We use $T_{\mathcal{S}}$ as a set of reference tetrahedra to which the tetrahedra of $\mathcal{T}$ should fit as well as possible. Consequently, the data attachment energy term we use simply measures the overlap between $T_{\mathcal{S}}$ and the tetrahedra of the current simplicial complex that can be expressed as:

$$T_{\mathcal{T}} = \left\{\mathcal{B}_3^3(t), t \in \mathcal{C}_3\right\}$$

The overlap is computed as a Jaccard index, with a negative sign since we want to maximize it:

$$E_{image}(\mathcal{T}, \mathcal{S}) = -\frac{|T_{\mathcal{T}} \cap T_{\mathcal{S}}|}{|T_{\mathcal{T}} \cup T_{\mathcal{S}|}} \tag{2}$$

The prior energy term aims to make the number of neighbors of the cells in the adjacency complex consistent with biological observations. Based on expertized tissue images, we remarked that the number of neighbors of an epidermis cell was 9.0 ± 2.1 and 13.0 ± 2.7 for an inner cell. Consequently, we set optimal values $N_{epi} = 9$ and $N_{inn} = 13$ and define the prior energy using the distance of the number of neighbors of each vertex to its ideal value (depending whether it touches the exterior or not):

$$E_{prior}(\mathcal{T}) = \frac{1}{|\mathcal{C}_0|} \sum_{v \in \mathcal{C}_0 \setminus v_{ext}} \left(|\mathcal{N}_0^+(v)| - N(v)\right)^2 \tag{3}$$

$$\forall v \in \mathcal{C}_0 \setminus v_{ext}, N(v) = \begin{cases} N_{epi} + 1 & \text{if } v_{ext} \in \mathcal{N}_0^+(v) \\ N_{inn} & \text{otherwise} \end{cases}$$

Finally, the last term considers the geometrical regularity of the elements of the complex. It tries to improve the quality of the tetrahedra in a way that fits the goal of recreating a plausible cell adjacency. Therefore, we defined it on the tetrahedra of $\mathcal{T}$, trying to avoid that links appear between tissue cells that lie too far from each other by measuring the maximal edge length of each tetrahedron:

$$E_{regularity}(\mathcal{T}) = \frac{1}{|\mathcal{C}_3|} \sum_{t \in \mathcal{C}_3} \max_{\substack{e \in \mathcal{B}_3^2(t) \\ \{v_1, v_2\} = \mathcal{B}_1(e)}} \|\mathcal{P}(v_1) - \mathcal{P}(v_2)\| \tag{4}$$

The energies having different, sometimes antagonist effects, it is important to balance well their influence to get the best result. To obtain a satisfactory result, we ran our optimization method using a (handset) range of energy weights and evaluated the results using our quality estimators. We retained the ones providing the best average and highest minimal values of the estimators, and found out the values $\omega_{image} = 8.0$, $\omega_{prior} = 0.05$ and $\omega_{regularity} = 0.5$ to offer the best compromise for the optimization of the adjacency complex.

1.3 Dual vertex positioning

In the dualization process, the geometry is defined by the positioning of the vertices associated with each thetrahedron of the simplicial complex of adjacency. However, since the Delaunay constraint no longer holds after several passes of topological optimization, we have to set these positions carefully to avoid geometrical artifacts such as folding or face intersections. In a Voronoi diagram, the dual vertices are located at the centers of the circumscribed sphere of their respective primal tetrahedra. We started with this idea, computing these positions as a weighted barycenter of the positions of the tetrahedra vertices.

In the case one of the computed weights is negative, it means that the center will lie outside the tetrahedron, a situation that might generate the evoked geometrical problems. To avoid them we chose to constrain the vertex to remain inside its tetrahedron. Consequently, in such cases, we take out the vertex associated with the most negative weight, and compute the position as the center of the circumscribed circle of the triangle formed by the three remaining vertices, once again computed as a weighted barycenter. And if a weight is still negative (meaning the triangle has one obtuse angle) the corresponding vertex is ignored and the position set as the middle point of the two remaining vertices.

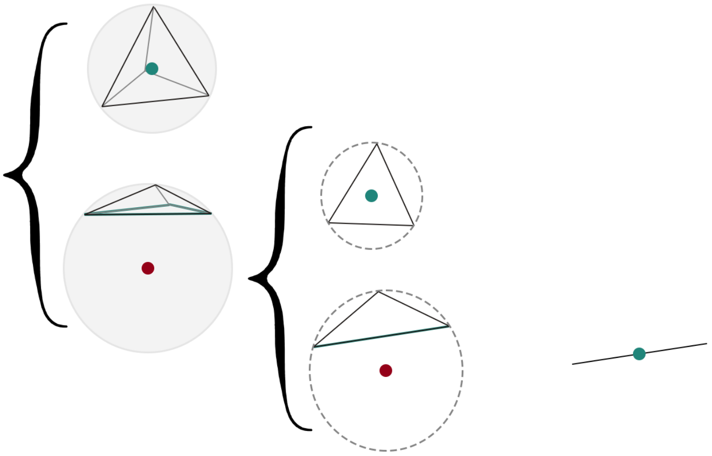

Figure 2. Computing the position of a dual vertex from a primal tetrahedron : the position is set to the circumscribed sphere center; if it lies outside of the tetrahedron, it is re-estimated as the circumscribed circle center of the nearest triangle; if it lies outside the triangle it is re-estimated as the middle point of the closest edge.

This process, illustrated in Figure 2 ensures that the position of the dual vertices will remain inside (or at worst on the border of) their primal tetrahedra and that no impossible geometric configurations will issue from the dualization process.

1.4 Dealing with the exterior in the dual reconstruction

The dualization of a simplicial complex poses a major problem relative to the boundaries of the considered object. We will illustrate this problem and the solution we proposed to solve it on a 2D example but it is general and extends very well in 3D. In our case, a simplical complex is constructed based on the adjacency relationships found in an image containing a limited number of finite cell regions as in Figure 3 (a). In a 2D case ($D = 2$), this allows us to define triangles linking vertices representing cell centers, triangles delimited by edges in such way that they form a cellular complex implemented as described in 1.1.

But among those edges (resp. triangles if $D = 3$), some play an important role: the ones that are linked to only one simplex of dimension D and that can be called exterior simplices:

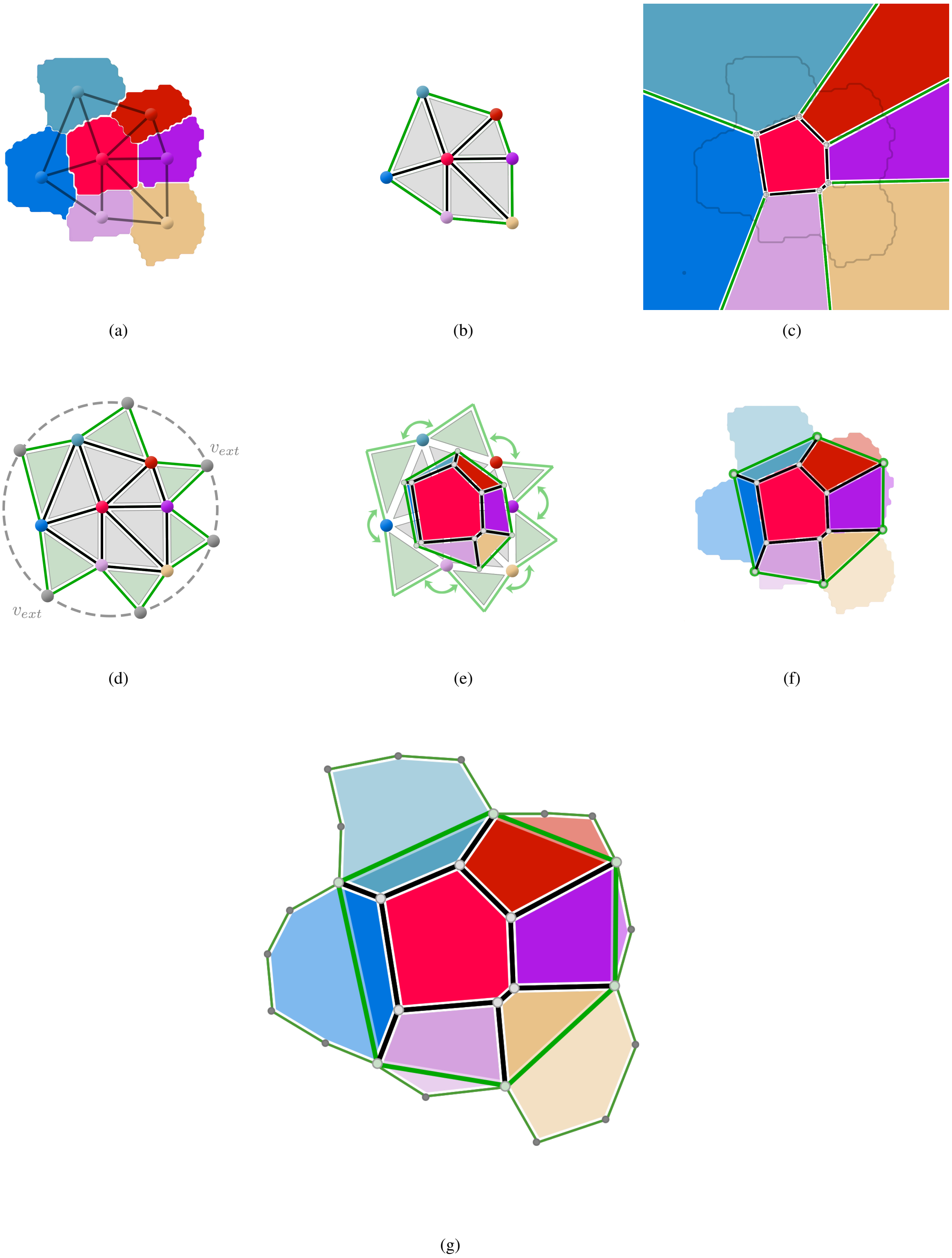

Figure 3. Representing the exterior for the dualization of a 2D simplicial complex: adjacency information extracted from a segmented image (a) and the resulting simplicial complex with exterior edges marked in green (b): in the dual, exterior edges correspond to infinite edges giving rise to infinite cells (c). Adding an artificial exterior vertex to the simplicial complex (d) allows to perform a dualization without infinite cells (e) but using the image is necessary to place new vertices accurately (f). With such topologically correct reconstruction, it is easier to reconstruct the object boundaries by subdivision and vertex relocation than if we had to compute intersection of a surface with infinite cells (g).

$$\mathcal{C}_{D-1}^{ext} = \{c \in C_{D-1} \mid |R_{D-1}(c)| = 1\}$$

The exterior edges form the boundary of the simplicial complex (Figure 3 (b)) and are theoretically dualized into an infinite edge issuing from the dual vertex corresponding to their unique triangle. These infinite dual edges define infinite cells in the dual cellular complex representing the tissue geometry as shown in Figure 3 (c). This raises important issues, the first one being how to represent infinity it the cellular complexes as we implement them? Should infinite edges be linked to only one vertex, or should a (unique) infinite vertex be introduced? And if we settle for ”very distant vertices” to limit the edges, positioned according to a fixed rule (following the edge bisector as in a Voronoi diagram for instance) the resulting cells would still be open, as no topological structure would be here to delimit them. It would then be necessary to compute this cell boundary element as the intersection of the open cell with the object's surface, a geometrical operation that is not trivial at all.

For all these reasons, we introduced an alternative approach to handle the case of exterior simplices to solve the problem in a more generic and elegant way. It relies on the addition in the adjacency simplicial complex of a virtual vertex v_{ext} representing the whole exterior region. Unlike the rest of the vertices that correspond to cell centers, $\mathcal{P}(v_{ext})$ is not defined, meaning the vertex has no fixed position in space. However it needs to comply with all the topological constraints regarding simplicial complexes. Its topological relationship to the rest of the complex are defined quite simply:

- $\forall c \in \mathcal{C}_{D-1}^{ext}$, a D-dimensional simplex c' is added so that $\mathcal{B}_D^D(c') = \mathcal{B}_{D-1}^{D-1}(c) \bigcup \{v_{ext}\}$
- All the missing simplices of dimension $< D$ forming the boundary of c' are also added to the complex

An example of this extension is given if the Figure 3 (d). First v_ext is added with no fixed spatial position. For each exterior edge e of the original simplicial complex, we add a new ”exterior” triangle linking the two vertices of the edge $v_1, v_2 = \mathcal{B}_1^1(e)$ and v_{ext}. In addition, the two edges linking respectively v_1 to v_ext and v_2 to v_ext are also added to the complex. This way, the triangles corresponding to adjacent exterior edges $e, e' \mid e' \in \mathcal{N}_1^-(e)$ will share a new edge in common: the one linking v_{ext} to $v = \mathcal{B}_1^1(e) \bigcap \mathcal{B}_1^1(e')$. This is of course difficult to visualize since v_{ext} has no position but as suggests Figure 3 (e), the new exterior simplices actually form a closed connected ”ring” of D-dimensional simplices around the initial complex.

The magic of this trick resides in the fact that now, all $D-1$-simplices are linked to exactly 2 D-simplices, therefore no infinite edge will appear in the dualization. Plus, the cell outer boundaries that had no topological existence in the previous case now correspond to the dual of the new edges linking cells to the exterior. The only problem is now a geometrical one: where to place the dual vertices of the new exterior D-simplices? In the general case, vertices of the geometry cellular complex are placed using the positions of the $D+1$ points forming their dual D-simplex in the adjacency complex, following a Voronoi-like rule: the center of circumscribed circle for a triangle, or sphere for a tetrahedron. But in the case of adjacency simplices containing v_{ext}, only D positions are known. Our solution was to do the same as if we were in a lower dimension: the middle point of the non-v_{ext} segment for an exterior triangle, the center of the circumscribed circle of the non-v_{ext} triangle for an exterior tetrahedron. This dual reconstruction process is the one used in Figure 3 (e).

However, these new vertices have a physical existence in the segmented image we start from: they are the points where the regions of the two cells (resp. three in the 3D case) forming part of the exterior edge meet with the exterior region. Their spatial position can generally be extracted from the image, and the new vertices can consequently be relocated to their actual position easily, without any other geometrical

computation. The result, illustrated in Figure 3 (f), is a more realistic cell geometry, even though cell shapes are limited by the fact that their exterior boundary consists of one single edge. To solve this accuracy problem, it is necessary to subdivide the cell boundaries, creating new vertices which positions can be optimized to fit better the shape of the object (for instance by projecting them onto the cell surface as in Figure 3 (g)).

1.5 Energy definition for tissue mesh optimization

The STEM component provides tools to optimize a 3D triangular mesh representing the cell tissue along several competing criteria. The mesh is represented as a cellular complex $\mathcal{S}$ composed of vertices, edges, triangular faces, and cells with an arbitrary number of faces. The optimization is here again performed as an iterative energy minimization process taking into account the consistency of the mesh with the segmented image $\mathcal{S}$, the shape of the individual cells, and the regularity of the mesh elements. Similarly to the one used for the adjacency complex optimization, the energy minimized here is composed of three terms:

$$E(\mathcal{M}, \mathcal{S}) = E_{image}(\mathcal{M}, \mathcal{S}) + E_{prior}(\mathcal{M}) + E_{regularity}(\mathcal{M}) \tag{5}$$

The first data attachment term has the objective of tying the vertices of the mesh (which represents cell boundaries as a complex of triangles) to actual cell interfaces in the image. Cell interfaces correspond to transitions between differently labeled regions in the image, something that is typically captured by a gradient operator in image analysis. But since the values in $\mathcal{S}$ are label identifying cells, and not signal values, we compute the magnitude of a pseudo-gradient capturing only the information of label differentiation without taking into account their numerical values. At each voxel position $\mathbf{x}$, it considers the number of different labels found in a neighborhood of radius σ; the obtained image is then filtered by a Gaussian operator to non-zero values in regions surrounding actual boundaries.

$$\|\nabla \mathcal{S}(\mathbf{x})\| = \begin{cases} 2\left|\mathcal{S}\big(\mathcal{N}_\sigma(\mathbf{x})\big)\right| & \text{if } 1 \in \mathcal{S}\big(\mathcal{N}_\sigma(\mathbf{x})\big) \\ \left|\mathcal{S}\big(\mathcal{N}_\sigma(\mathbf{x})\big)\right| & \text{otherwise} \end{cases} * N(\cdot, \sigma) \tag{6}$$

The high values of $\|\nabla \mathcal{S}\|$ therefore correspond to places where cells meet, which is where we want the mesh vertices to end up. The image attachment energy term should therefore maximize the pseudo-gradient magnitude of the mesh vertices:

$$E_{image}(\mathcal{M}, \mathcal{S}) = \omega_{gradient} E_{gradient}(\mathcal{M}, \mathcal{S}) = \omega_{gradient} \sum_{v \in \mathcal{C}_0} -\|\nabla \mathcal{S}\left(\mathcal{P}(v)\right)\| \tag{7}$$

The prior energy term is here to ensure that the shape of the cells will correspond to what we consider a desirable outcome: convex volumes with convex polygonal faces. The energy combines those two aspects under the form of two weighted energy terms:

$$E_{prior}(\mathcal{M}) = \omega_{planarity} E_{planarity}(\mathcal{M}) + \omega_{contour} E_{contour}(\mathcal{M}) \tag{8}$$

The first one aims at making the cell interfaces flat, so that no concavities form between two cells. It is minimal when the overall distance of the vertices belonging to the interface between cells c and c' to its

mean plane (represented by the interface barycenter $\overline{\mathcal{P}}_{c,c'}$ and average normal vector $\overline{\vec{n}}_{c,c'}$) is minimal:

$$E_{planarity}(\mathcal{M}) = \sum_{v \in \mathcal{C}_0} \Big(\sum_{c \in \mathcal{R}_0^3(v)} \sum_{\substack{c' \in \mathcal{R}_0^3(v) \\ c' \neq c}} \Big| \left(\mathcal{P}(v) - \overline{\mathcal{P}}_{c,c'}\right) \cdot \overline{\vec{n}}_{c,c'} \Big| \Big) \tag{9}$$

The second one tries to make the cell edges straight to end up with simple polygonal cell interfaces. It is based on the laplacian operator that is used to reduce the noise on the linear cell boundaries:

$$E_{contour}(\mathcal{M}) = \sum_{v \in \mathcal{C}_0} \Big(\sum_{c \in \mathcal{R}_0^3(v)} \sum_{\substack{c' \in \mathcal{R}_0^3(v) \\ c' \neq c}} \left\| \Delta \mathcal{C}_{c,c'}\left(\mathcal{P}(v)\right) \right\| \Big) \tag{10}$$

And finally, the regularity term ensures that the quality of the triangles remains satisfactory enough in spite of the effects of the two other energies that apply without taking the shape of the mesh elements into account. Here we consider mesh quality as the regularity of the mesh triangles both in size and in shape, which leads again to a decomposition of the energy into two terms:

$$E_{regularity}(\mathcal{M}) = \omega_{area} E_{area}(\mathcal{M}) + \omega_{eccentricity} E_{eccentricity}(\mathcal{M}) \tag{11}$$

The first one ensures the global similarity in size of all the triangles of the mesh. It should be minimal when all the triangles have the same area, and therefore is based on the squared deviation of triangle areas to the average triangle area over the mesh noted $\bar{A}$:

$$E_{area}(\mathcal{M}) = \sum_{v \in \mathcal{C}_0} \frac{1}{3} \sum_{t \in \mathcal{R}_0^2(v)} \left(A\left(\mathcal{P}\left(\mathcal{B}_2^2(t)\right)\right) - \bar{A} \right)^2 \tag{12}$$

The second one pushes triangle to be as regular as possible, meaning it should be minimal when all triangles are equilateral. Measures of ”how equilateral” a triangle is are generally called eccentricity of a triangle, with an equilateral triangle having an eccentricity of 0; consequently we want to minimize the global eccentricity of the mesh triangles. A convenient measure of triangle eccentricity (easy to compute from triangle edge lengths, and with good differentiability properties) involves the sum of the sinuses of a triangle’s angles:

$$E_{eccentricity}(\mathcal{M}) = \sum_{v \in \mathcal{C}_0} \frac{1}{3} \sum_{t \in \mathcal{R}_0^2(v)} 1 - \frac{2}{3\sqrt{3}} \sum_{v \in \mathcal{B}_2^2(t)} \sin\left(\widehat{\mathcal{P}(v)}\right) \tag{13}$$

The optimization takes the form of an energy gradient descent of the positions $\mathcal{P}$ of the mesh vertices, and at each iteration the direction in which to move each vertex is computed as the weighted sum of the local gradient of each energy involved at the current position of the point. Similarly to what was done for the adjacency complex optimization energy weights, we found that the best empirical values for these weights were $\omega_{gradient}$ = 0.17, $\omega_{planarity}$ = 0.47, $\omega_{contour}$ = 1.3, $\omega_{eccentricity}$ = 2.0 and ω_{area} = 0.005.

2 IMPLEMENTATION DETAILS

As a part of the open-source plant modelling library OpenAlea (Pradal et al., 2008), the DRACO-STEM package is a pure Python package designed to work on common data structures, usable by the other packages of the library. The dependencies are limited to the core components of the OpenAlea framework (`https://github.com/openalea/openalea-components`), the specific data structures and algorithms designed to manipulate cellular complexes (`https://github.com/VirtualPlants/cellcomplex`) as well as widespread scientific programming Python libraries NumPy (Walt et al., 2011) and SciPy (Jones et al., 2001–).

2.1 Data Structures

OpenAlea provides a data structure to represent 3D image stacks such as those obtained from a confocal microscope. This structure called **`SpatialImage`** basically contains the image matrix as a three dimensional NumPy array as well as some required metadata such as the image resolution (or physical size of the voxels, along the three axes of the image). Readers are provided for various file formats (**`.tif`**,**`.lsm`**,**`.inr`**) making it easy to import images obtained from whatever source. Images of segmented cell tissue are supposed to be 8-bit or 16-bit integer images, in which the cell regions have a unique label and the background region a label set to 1.

Concerning cellular complexes, the implementation as an incidence graph detailed in Section 2.2 is realized by a structured called **`PropertyTopomesh`** which gives the possibility to contain (in addition to the topological relationships between elements of dimension 0, 1, 2 and 3) properties defined on each of those elements. Examples of such properties are numerous, they could be the position of a vertex given by its 3D coordinates, the length of an edge, the curvature tensor estimated on a triangular face, of the volume of a cell. Any user defined property can be added to the structure as a simple Python dictionary. Such structures, along with their properties (or at least a subset of them) can be exported to the common mesh file format **`.ply`** following a standard allowing to reconstruct the topological structure (Krupinski et al., 2015), for the same motive of compatibility with other mesh viewing software or modeling environments.

2.2 Code Examples

In the DRACO-STEM package, all functionalities are provided by a high-level object of the class **`DracoMesh`** encompassing the input image as a **`SpatialImage`**, the adjacency relationships extracted from it, the constructed adjacency complex as a **`PropertyTopomesh`** and the dual reconstructed also as a **`PropertyTopomesh`**. The methods allowing to perform the different steps of the pipeline have to be called in the right order, but have several editable parameters on which the user can play.

As shown in Table 1, the **`DracoMesh`** is initialized using a **`SpatialImage`** that can be loaded using the provided reader **`imread`**. It could also be passed as a filename, and it is also possible to specify a path to previously extracted adjacency simplices to speed up the initialization step (if the file is not existing, the computed information will be saved anyways for further use).

The first method to call is then the one initializing the adjacency complex by the Delaunay tetrahedrization of the cell barycenters. This method called **`delaunay_adjacency_complex`** also performs the surface triangle removal step, necessary to obtain a concave Delaunay tetrahedrization. To do so, it takes as argument the criteria used to determine which external triangles should be removed, as a list containing string among the following ones:

- **`'surface'`**: remove triangles intersecting the external surface of the tissue object .

Table 1. Example of the use of DRACO-STEM within the OpenAlea framework: opening an image, launching the two-step adjacency complex optimization with custom parameters, and generating (and saving) the dual geometry mesh.

```
import openalea.draco_stem
from openalea.draco_stem.draco.draco import DracoMesh

from openalea.image import SpatialImage
from openalea.image.serial.all import imread

from openalea.mesh import PropertyTopomesh
from openalea.mesh.property_topomesh_io import save_ply_property_topomesh

from openalea.deploy.shared_data import shared_data
dirname = shared_data(openalea.draco_stem)
filename = "example_seg"
inputfile = dirname+"/segmented_images/"+filename+".inr.gz"
img = imread(inputfile)

draco = DracoMesh(image=img)

draco.delaunay_adjacency_complex(
        surface_cleaning_criteria=['surface', 'sliver', 'distance'])
draco.adjacency_complex_optimization(
        omega_energies=dict(image=8.0, adjacency=0.05, geometry=0.5),
        n_iterations=5)

triangular= ['star', 'remeshed', 'projected', 'exact', 'flat']
dual_topomesh = draco.dual_reconstruction(
        reconstruction_triangulation=triangular,
        adjacency_complex_degree=3)

topomesh_filename = dirname+"/output_meshes/"+filename+"_topomesh.ply"
save_ply_property_topomesh(dual_topomesh, topomesh_filename)
```

- `'distance'`: remove triangles linking cells that are too distant from each other.
- `'sliver'`: remove triangles forming flat tetrahedra with non-degenrated faces (known as slivers).

Then the core of the DRACO method is the optimization of this adjacency complex, which is carried out by the method **adjacency_complex_optimization**. Here again, it is possible to tune the number of iterations in the simulated annealing optimization process, as well as the weights of the different energies for the optimization defined in Equation 1. Those weights are specified in a Python dictionary for which the keys are `'image'` for ω_{image}, `'neighborhood'` for ω_{prior} and `'geometry'` for $\omega_{regularity}$. Running this method updates the **PropertyTopomesh** attribute of the **DracoMesh** object that represents the adjacency simplicial complex.

Finally, once the adjacency complex has been optimized, the last method aims at reconstructing the triangular mesh representing the geometry of the tissue. **dual_reconstruction** does exactly just that, dualizing the adjacency into a new **PropertyTopomesh**. But the user can control some features of the returned mesh, notably on the way the cell interfaces are triangulated, and on how the energies of the STEM component should be balanced. Again this has to be set at high level by passing a list of instructions among the following ones:

- `'star'` or `'delaunay'`: initial triangulation of polygonal interfaces.
- `'split'` or `'remeshed'`: subdivision of triangles (global, or by local topological operations).
- `'projected'`: projection of the surface triangles on the surface of the tissue object.
- `'regular'` or `'realistic'` or `'exact'`: degree of mesh smoothing / cell shape exactness (STEM).
- `'flat'` or `'straight'`: constraints on the shape of interfaces or interface edges (STEM).

Once obtained the dual mesh through this method, it can then be exported to a convenient format using **save_ply_property_topomesh**, and be used for visualization in OpenAleaLab Coste et al. (2014) or other compatible software, or for any geometry-based simulation in an other modeling environment.

REFERENCES

Cerutti, G. and Godin, C. (2015). Meshing meristems - an iterative mesh optimization method for modeling plant tissue at cell resolution. In *BIOIMAGING*

Cerutti, G., Ribes, S., Galvan-Ampudia, C., Vernoux, T., and Godin, C. (2015). 3D tessellation of plant tissue - A dual optimization approach to cell-level meristem reconstruction from microscopy images. In *International Conference on 3D Vision*. 443–451

Coste, J., Baty, G., Boudon, F., , Godin, C., and Pradal, C. (2014). OpenAleaLab: An integrated multi-paradigm modelling environment. In *EuroScipy 2014*

Jones, E., Oliphant, T., Peterson, P., et al. (2001–). SciPy: Open source scientific tools for Python. http://www.scipy.org/

Krupinski, P., Jönsson, H., Tauriello, G., Smith, R. S., and Barbier de Reuille, P. (2015). Exchange format for geometries. In *Sainsbury Computational Biology Workshop*

Pradal, C., Dufour-Kowalski, S., Boudon, F., Fournier, C., and Godin, C. (2008). OpenAlea: a visual programming and component-based software platform for plant modelling. *Functional Plant Biology* 35, 751–760. https://github.com/openalea

Walt, S. v. d., Colbert, S. C., and Varoquaux, G. (2011). The numpy array: A structure for efficient numerical computation. *Computing in Science and Engineering* 13, 22–30. http://www.numpy.org
